# Differential Effects of Western and Mediterranean-Type Diets on Gut Microbiota: A Metagenomics and Metabolomics Approach

**DOI:** 10.3390/nu13082638

**Published:** 2021-07-30

**Authors:** Claudia Barber, Marianela Mego, Carlos Sabater, Fernando Vallejo, Rogger Alvaro Bendezu, Marcela Masihy, Francisco Guarner, Juan Carlos Espín, Abelardo Margolles, Fernando Azpiroz

**Affiliations:** 1 Digestive System Research Unit, University Hospital Vall d’Hebron, Centro de Investigación Biomédica en Red de Enfermedades Hepáticas y Digestivas (Ciberehd), 08035 Barcelona, Spain; claudiabarbercaselles@gmail.com (C.B.); marianelamego@hotmail.com (M.M.); alvarobendezu@hotmail.com (R.A.B.); marcelamasihy@gmail.com (M.M.); fguarner@icloud.com (F.G.); 2 Departament de Medicina, Universitat Autònoma de Barcelona, 08193 Bellaterra, Spain; 3 Department of Microbiology and Biochemistry, IPLA-CSIC, 33300 Asturias, Spain; carlos.sabater@csic.es (C.S.); amargolles@ipla.csic.es (A.M.); 4 Health Research Institute of Asturias, ISPA, 33011 Asturias, Spain; 5 Metabolomics Service, CEBAS-CSIC, 30100 Murcia, Spain; fvallejo@cebas.csic.es; 6 Laboratory of Food & Health, Group of Quality, Safety, and Bioactivity of Plant Foods, CEBAS-CSIC, 30100 Murcia, Spain; jcespin@cebas.csic.es

**Keywords:** Western-type diet, Mediterranean-type diet, gut microbiota, metagenomics, metabolomics, digestive sensations, intestinal gas

## Abstract

Our aim was to determine the effect of diet on gut microbiota, digestive function and sensations, using an integrated clinical, metagenomics and metabolomics approach. We conducted a cross-over, randomised study on the effects of a Western-type diet versus a fibre-enriched Mediterranean diet. In 20 healthy men, each diet was administered for 2 weeks preceded by a 2-week washout diet. The following outcomes were recorded: (a) number of anal gas evacuations; (b) digestive sensations; (c) volume of gas evacuated after a probe meal; (d) colonic content by magnetic resonance imaging; (e) gut microbiota taxonomy and metabolic functions by shotgun sequencing of faecal samples; (f) urinary metabolites using untargeted metabolomics. As compared to a Western diet, the Mediterranean diet was associated with (i) higher number of anal gas evacuations, (ii) sensation of flatulence and borborygmi, (iii) larger volume of gas after the meal and (iv) larger colonic content. Despite the relatively little difference in microbiota composition between both diets, microbial metabolism differed substantially, as shown by urinary metabolite profiles and the abundance of microbial metabolic pathways. The effects of the diet were less evident in individuals with robust microbiotas (higher beta-diversity). To conclude, healthy individuals tolerate dietary changes with minor microbial modifications at the composition level but with remarkable variation in microbial metabolism.

## 1. Introduction 

Previous studies in our and other laboratories have shown that diet influences both the gut microbiota’s metabolic activity and composition [1]. The amount of intestinal gas production can evaluate intestinal microbiota’s metabolic activity as an indirect parameter associated with abdominal symptoms. Recently developed shotgun sequencing technologies and bioinformatic analysis of sequences can assess the microbial composition and the gut microbiota’s metabolic potential in DNA extracts from faecal samples [2]. Moreover, untargeted urine metabolomics has emerged as a valuable tool to detect subtle metabolic changes related to the gut microbiota metabolism, which can be influenced by the diet [3]. 

On the other hand, a series of studies have shown that a meal produces a digestive response that also involves conscious sensations such as satiety/fullness (homeostatic sensations) and digestive well-being/mood (hedonic sensations). Homeostatic and hedonic sensations are dissociable; hence, postprandial satiety may have either a positive or a negative hedonic dimension (postprandial satisfaction or dissatisfaction), depending on a series of conditioning factors. We hypothesised that the diet influences gastrointestinal physiology, intestinal microbiota and the responses to a comfort meal. Therefore, we aimed at comparing the effects of two diets (Western-type and Mediterranean-type diets) on gastrointestinal physiology, microbiota composition and functionality and the responses to a comfort meal. To this purpose, we used an integrated clinical metagenomics, and metabolomics approach.

## 2. Material and Methods 

### 2.1. Study Design

**Figure 1:**
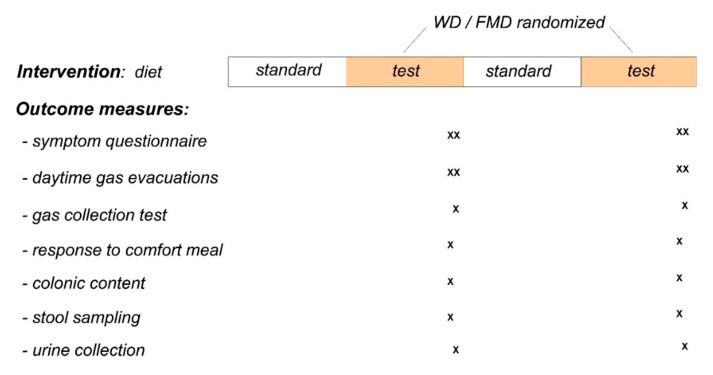
Experimental design. Cross-over, randomised study; 20 participants were included and 18 adhered to study instructions and were included for analysis.

Our study was a single-centre, cross-over, randomised, open-label study comparing the effect of a high-fat, low-residue diet (Western-type diet; WD) versus a low-fat, high-residue diet (fibre-enriched Mediterranean diet; FMD). Each diet was administered for 2 weeks consecutively in randomised order, and each period was preceded by 2 weeks of a balanced diet; the total duration of the study was 8 weeks. Outcomes were measured during the last 2 days of each diet period (Figure 1). The research was conducted according to the Declaration of Helsinki. The protocol for the study had been previously approved by the Institutional Review Board of the University Hospital Vall d’Hebron, (Comitè d’Ètica d’Investigació Clinica, Vall d’Hebron Insititut de Recerca; protocol number PR(AG)338/2016 approved 28 October 2016) and all participants provided written informed consent. The protocol was registered with ClinicalTrials.Gov [NCT03783221].

### 2.2. Participants

Twenty healthy volunteers without gastrointestinal symptoms or history of gastrointestinal disorders participated in the study. For the sake of homogeneity, only men were recruited. All participants were instructed to fill out a clinical questionnaire based on Rome IV criteria to rule out functional gastrointestinal disorders (no symptom ≥ 2 on a 0–10 scale) and confirm normal bowel habits. This questionnaire has been previously shown to discriminate patients from healthy subjects [1,4,5,6,7,8]. Antibiotic, prebiotic or probiotic consumption during the previous 2 months was an exclusion criterium, and if necessary, the study entry was postponed to match this requirement. 

### 2.3. Intervention 

The high-residue FMD contained 19% fat, 62% carbohydrates and 16% proteins with 54.2 g fibre. The high-fat WD contained 51% fat, 27% carbohydrates and 21% proteins with 4.7 g fibre. The washout diet (23% fat, 55% carbohydrates, 22% proteins) was administered two weeks before the high-residue and the high-fat diets. Participants were given detailed diet plans (see Supplementary Material: Detailed Diets), and each week, they came to the clinical study unit to pick up the specific foodstuffs in precise serving portions to be consumed. They were instructed to mark in the daily diet plan the items not completely consumed (about half of the indicated portion or not at all). Based on the fibre and fat content in each item, adherence to the diets was calculated and expressed as % of fibre consumed of the amount indicated in the FMD and % of fat consumed of that indicated in the WD. Participants were instructed to avoid dietary beverages, and a maximum of 10 g alcohol per day was allowed, but intake of water and other beverages was not monitored. All the participants were asked to avoid during the study the ingestion of any fermented dairy products (yoghurts with living strains or probiotics containing products) and any tablet, pill or food supplement containing pre- or probiotics (Figure 1). Dietary instructions were reinforced at each visit to assure adherence to the study. Participants fulfilled daily questionnaires specifying the items consumed in less than 50% of the amount indicated in the diet. 

### 2.4. Outcomes

During the last 2 days on WD and FMD (evaluation periods), the following outcomes were measured.

#### 2.4.1. Daily Symptom Questionnaire and Bowel Habit

During the last 2 days of each evaluation period, the participants were instructed to fill out daily questionnaires (Figure 1) that included the following parameters: (a) subjective sensations of flatulence (defined as anal gas evacuation), abdominal bloating (pressure/fullness), abdominal distension (sensation of girth increment), borborygmi and abdominal discomfort/pain using 0–10 analog scales; (b) digestive well-being using a 10-point scale graded from +5 (extremely pleasant sensation/satisfaction) to −5 (extremely unpleasant sensation/dissatisfaction) and mood on similar scale graded from +5 (very positive) to −5 (very negative); (c) the number of bowel movements; (d) stool form using the Bristol stool form scale; and (e) stool weight. Participants were provided with a balance (digital weighing scale BT-32013, El Corte Ingles, Madrid, Spain) and received instructions to measure stool weight and to fill out the scales by the end of the day. This questionnaire has been previously used and was shown sensitive to detect effects of dietary interventions in different populations [1,4,8,9,10,11,12].

#### 2.4.2. Number of Anal Gas Evacuations 

During the last 2 days of each evaluation period, the number of anal gas evacuations was measured using an event marker (Hand Tally Counter No 101, Digi Sport Instruments, Shangqiu, China). Participants were instructed to carry the event marker during the day and register each passage of anal gas (Figure 1). This method has been previously used with reproducible and consistent results [1,4]; furthermore, studies measuring the number of gas evacuations by an event marker and continuously recording anal gas evacuations have shown an excellent correlation (R > 0.95; *p* < 0.05) [13,14,15,16].

#### 2.4.3. Intestinal Gas Production

On the last day of each evaluation period, intestinal gas production after a probe meal was measured (Figure 1). Participants reported to the laboratory in the morning after an overnight fast and consumed a probe meal consisting of 300 g stewed beans (Fabada Asturiana, Litoral, Barcelona, Spain) and 200 mL water (426 Kcal, 25 g fat, 19 g protein, 24 g carbohydrates, 16 g fibre). Based on preliminary studies, participants were instructed to consume the meal in 12 min at a comfortable eating rate. 

As previously described, the volume of gas evacuated per anus was measured for 4 h after the probe meal [1,17,18]. In brief, gas was collected using a rectal balloon catheter (20 F Foley catheter, Bard, Barcelona, Spain) connected via a gas-tight line to a barostat, and the volume was continuously recorded. The intrarectal balloon was inflated with 5 mL of water to prevent anal gas leaks. 

#### 2.4.4. Response to a Comfort Meal 

On the first day of each evaluation period, the biological response to a comfort meal (sensations and digestive function) was tested (Figure 1). The comfort meal consisted of a freshly cooked, warm sandwich (58 g bread with 12 g butter, 38 g ham and 38 g cheese) and 200 mL orange juice (300 mL total volume, total caloric content of 425 Kcal; 17 g lipids, 47 g carbohydrates, 18 g proteins). Participants were instructed to consume the meal in 12 min at a comfortable eating rate. 

Digestive sensations were measured using graded scales, as follows. Two 10 cm scales graded from 0 (not at all) to 10 (very much) were used to measure abdominal fullness and discomfort/pain; four additional 10 cm scales graded from +5 to −5 were used to measure hunger/satiety (from extremely hungry to completely sated), digestive well-being (from extremely pleasant sensation/satisfaction to extremely unpleasant sensation/dissatisfaction) and mood (from very positive to very negative). Subjects received standard instructions on how to fill out the scales. The scales were scored before ingestion and at 0 min, 30 min and 60 min after ingestion. The method has been previously described in detail [19,20]. 

Antral and gallbladder emptying were measured by ultrasonography, as previously described in detail [8,9,10,12,21]. In brief, ultrasound images of the gastric antrum and the gallbladder were obtained using a Chison ultrasound scanner (ECO1; Chison, Wuxi, China) with an abdominal 3.5 Hz probe (C3A; Chison, Wuxi, China); images were obtained with the subjects seated and leaning slightly backwards in a chair. Gastric images between antral contractions were obtained in triplicate before, and at 0 min, 30 min and 60 min after the meal; using the superior mesenteric vein and the aorta as landmarks, the outer profile and the cross-sectional area of the antrum were measured using the built-in caliper and measurement tool. The longitudinal axis of the gallbladder was measured before and 60 min after the meal. 

#### 2.4.5. Colonic Content Measurement

During each evaluation period, colonic content was measured using magnetic resonance imaging (MRI) of the abdomen (without contrast administration) (Figure 1). Abdominal MRI scans were obtained using a 1.5 T MR imaging system (Aera; Siemens Healthcare, Erlangen, Germany). Analysis of the images was made using an original software developed for this purpose [22] (see Supplementary Material).

#### 2.4.6. Microbiota Composition 

On the first day of each evaluation period, faecal samples were collected, homogenised and immediately frozen by the participants in their home freezers at −20 °C (Figure 1). Samples were brought to the laboratory in a freezer pack, where they were stored at −80 °C. 

Genomic DNA extraction. A frozen aliquot (250 mg) of each sample was suspended in 250 µL of guanidine thiocyanate, 0.1 M Tris (pH 7.5), 40 µL of 10% *N*-lauroyl sarcosine and 500 µL 5% *N*-lauroyl sarcosine. DNA was extracted by mechanical disruption of microbial cells with beads, and recovery of nucleic acids from clear lysates was achieved by alcohol precipitation, as previously described [23]. An equivalent of 1 mg of each sample was used for DNA quantification using a NanoDrop Spectrophotometer (ND-1000, Nucliber, Madrid, Spain). 

Metagenomic analysis. After shotgun sequencing of DNA extracts, quality control of metagenomic samples was assessed by FastQC software (v0.11.9). Then, in silico separation of bacterial reads from contaminant reads was performed using the latest version of Kneaddata (v0.7.4) and Trimmomatic (v0.39) to discard low-quality sequences. The minimum length was computed as 50% of the length of the input reads. Sequencing reads were mapped by using Bowtie2 (v2.4.2) [23] against the reference databases “*Homo sapiens* hg37 and human contamination Bowtie2” (v0.1) to remove contaminations.

Taxonomic and functional analysis of microbial strains was performed using MetaPhlAn 2.0 (v2.9.14) and HUMAnN 2.0 (v2.9.0), respectively [24,25]. Taxonomic identification relies on clade-specific marker genes included in the “v296_ChocoPhlAn_201901” database. For functional analysis through HUMAnN 2.0 pipeline, reference protein database UniRef was used to profile the abundance of microbial gene families and metabolic pathways. Gene family and pathway abundances were re-normalised and expressed in units of copies per million.

Diversity estimators and similarity of the microbial communities between the samples was calculated by UniFrac method [26] and Phyloseq [27] package. Microbiome [28] package was also used in the analysis of sequencing data, including ordination analysis. Samples were clustered based on the Bray–Curtis dissimilarity metric to determine the stability of the microbiota of each participant. Participants whose samples at the two study periods (WD and FMD) clustered together were considered to have a robust microbiota.

#### 2.4.7. Metabolomic Analysis

On the last day of each evaluation period, the participants collected morning urine samples and immediately brought them to the laboratory. The samples were frozen for further analysis (Figure 1). 

Urine samples processing and UPLC-QTOF-MS analyses. As the pooling of 24 h volume urine was not feasible, creatinine was measured to allow diuresis standardisation. The urinary excretion of creatinine was determined as reported elsewhere [29]. Urine samples (500 μL) were centrifuged at 14,000× *g* for 5 min in a microcentrifuge at 4 °C. The supernatant was filtered through a 0.22 µm filter (Millipore, Billerica, MA, USA) and analysed (1 μL) by UPLC-ESI-QTOF-MS. In a second batch of experiments, urine samples were treated with glucuronidase and sulfatase as reported elsewhere [29] to remove glucuronide and sulphate moieties from the molecules (Supplementary Material). This approach was followed when authentic standards of glucuronide and sulphate conjugates were not commercially available, but standards for free forms were available.

Urine samples (with and without enzymatic treatment) were analysed by UPLC-ESI-QTOF-MS, as previously reported [30]. Briefly, the equipment consisted of an Agilent 1290 Infinity series LC system coupled to a 6550 I-Funnel Accurate-Mass QTOF (Agilent Technologies, Waldrom, Germany) with a dual electrospray ionisation interface (ESI-Jet Stream Technology, Waldbroon, Germany) for simultaneous spraying of a mass reference solution that enabled continuous calibration of detected *m*/*z* ratios (Supplementary Material). Feature extraction was carried out on Agilent Profinder B.06.00, a stand-alone feature extraction program for LC-MS-based profiling analyses (Supplementary Material). Determining compounds by molecular features (MFs) was carried out using a pre-filter to take peaks with a height greater or equal to 10,000 counts, allowing only -H and -HCOO as negative ions species and +H as positive ions (Supplementary Material).

Reagents. Propionyl-L-carnitine, deoxycholic acid, chenodeoxycholic acid, ursodeoxycholic acid, hyodeoxycholic acid, D-sphingosine, *p*-tyramine, 5-hydroxindole, L-aspartyl-L-phenylalanine, 3,4-dihydroxy-phenylpropionic acid, cortolone, trimethylamine-*N*-oxide (TMAO), formic acid, acetonitrile (ACN) and methanol (MeOH) were purchased from Sigma-Aldrich (Sigma-Aldrich, St. Louis, MO, USA), as well as β-glucuronidase (≥100,000 units/mL) and sulfatase (>10,000 units/g) from *Helix pomatia*.

### 2.5. Statistical Analysis

*Sample size calculation.* In a previous study, the number of daily anal gas evacuations during the administration of a low-residue diet decreased from 18 ± 2 evacuations before the diet to 11 ± 2 evacuations after 10 days of administration. Based on these data, it was estimated that a sample size of 20 subjects (paired) would detect a change in the daily number of anal gas evacuated (primary outcome) with 90% power and a 5% significance threshold. 

*Overall comparisons of physiological parameters.* The means (±SE) of the variables measured were calculated. The Kolmogorov–Smirnov test was used to check the normality of the data distribution. Parametric normally distributed data were compared by Student’s *t*-test for paired data; otherwise, the Wilcoxon signed-rank test was used. Comparison of gas volumes evacuated after the probe meal was performed after logarithmic conversion. 

*Responses to the comfort meal*. Temporal responses to the ingestion of the comfort meal and the effect of the diet were analysed using a repeated measures ANCOVA (dependent variable: post-prandial sensations scores; between and within subject factors: diet and time, respectively; covariate: pre-meal scores). This analysis was computed on IBM SPSS Statistics for Windows, Version 25.0. (IBM Corp., Armonk, NY, USA).

*Metagenomic data.* After the assignment of reads to species and strain level, DESeq2 differential abundance testing for sequencing data [31] was performed. p_adj_ values lower than 0.05 and log2 fold-change greater than 1.5 were considered to determine statistical differences in taxonomic and gene data according to the intervention (WD or FMD) or the stability of the microbiota (robust or non-robust). Statistically significant clades, gene families and metabolic pathways were associated with clinical parameters and metabolic profiles of participants through hierarchical all-against-all association testing (HAIIA) (huttenhower.sph.harvard.edu/halla last accessed on 19 November 2020) [32]. Pairwise Pearson coefficients were calculated considering *q*-values and a Bonferroni false discovery rate of 0.05. Moreover, multi-omic data integration using the DIABLO biomarker discovery pipeline [33] was used to determine characteristic fermentation profiles. All statistical tests were computed on R v3.5.0.

Metabolomic data. The final files were exported into the Mass Profiler Professional (MPP) 2.0 software package (Agilent, Santa Clara, CA, USA) for statistical analysis. Student’s *t*-test unpaired analyses were applied with a significance level of *p* < 0.05 with Bonferroni Holm Family-wise Error Rate (FWER) multiple testing corrections and a fold-change cut-off of 2.0-fold. Unidentified compounds were aligned across the different samples based on their retention times’ tolerance and the mass spectral similarity. The MassHunter MSC (Molecular Structure Correlator) program was used to correlate accurate mass MS/MS fragment ions for a compound of interest with one or more proposed molecular structures for that compound (Supplementary Material).

## 3. Results 

### 3.1. Demographics and Study Flow

Twenty healthy men (age range, 18–38 years old; 19.2–25.5 kg/m^2^ body mass index range) were included in the study; 18 of them adhered to study instructions, completed the protocol and were included for analysis. Analysis of the daily questionnaires specifying food consumption showed that 16 of the 18 participants consumed over 90% of the amount of fibre indicated in the FMD; the other two consumed 88% and 75% of the fibre indicated, respectively, but showed no differences in the gas evacuation pattern (see below) as compared to the rest. On the WD, 17 participants consumed over 90% and one consumed 88% of the amount of fat indicated. 

### 3.2. Clinical Parameters: Sensations and Bowel Habit 

**Figure 2:**
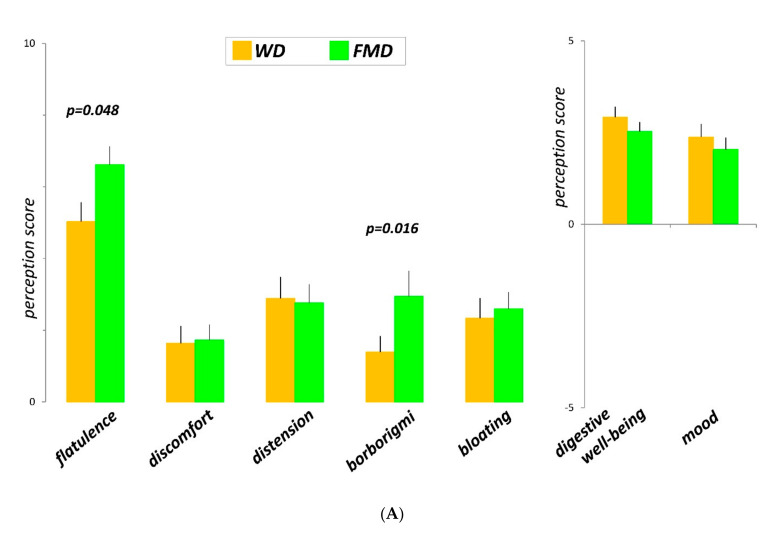

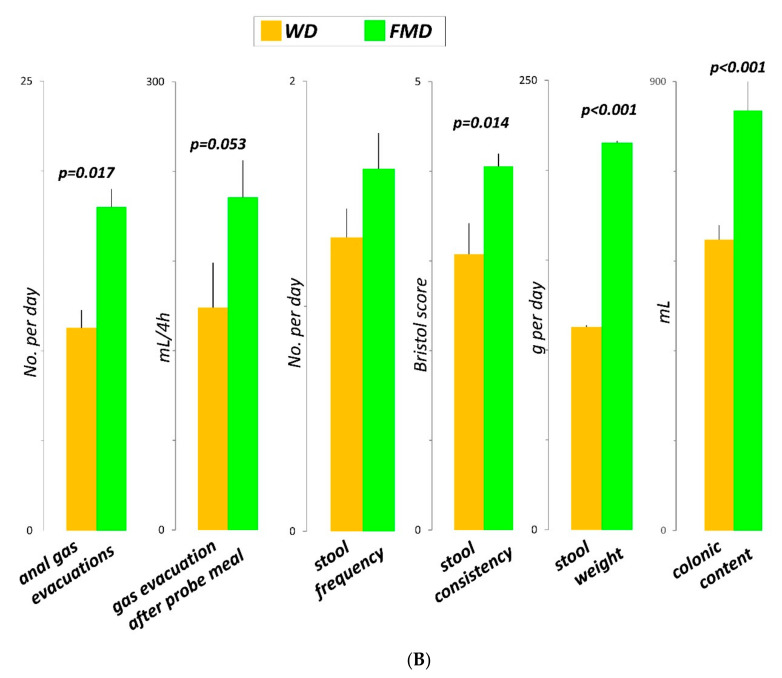
(**A**) Sensations measured by daily questionnaires during the last 2 days of the evaluation periods (*n* = 18). Data are average of 2 consecutive days. (**B**) Physiological parameters measured during the last 2 days of the evaluation periods. Values of number of daytime anal gas evacuations, number of bowel movements, Bristol stool form scale and faecal weight are average of 2 consecutive days; volume of gas evacuated during 4 h after a probe meal, and colonic content measured only once (*n* = 18).

Both diets were well tolerated with a sensation of digestive well-being and positive mood. The FMD was associated with significantly higher scores of sensation of flatulence and borborygmi than the WD without differences in the rest of the sensations reported (Figure 2A). As compared to the WD, the FMD was associated with more stool output and softer stool consistency, without differences in the number of daily bowel movements (Figure 2B). 

### 3.3. Number of Anal Gas Evacuations

Participants marked significantly more anal gas evacuations per day on the FMD than on the WD (Figure 2B). 

### 3.4. Volume of Gas Evacuated after the Probe Meal

The volume of gas evacuated during 4 h after the probe meal was more significant on the FMD than on the WD (Figure 2B). 

### 3.5. Responses to the Comfort Meal

Ingestion of the comfort meal induced satiety, mild fullness and digestive well-being without discomfort or changes in mood. No differences in the responses to ingestion were detected when the meal was consumed during the WB or the FMD periods. Meal ingestion produced a significant increase in the antral cross-section (reflecting gastric filling), followed by a gradual decrease during the postprandial period (reflecting gastric emptying). Concomitantly, meal ingestion produced a significant reduction in gallbladder longitudinal axis, reflecting gallbladder emptying. No differences in the gastric and gallbladder responses to the meal consumed during the WD and FMD were detected (data not shown).

### 3.6. Colonic Content

The total volume of colonic content was significantly higher on the FMD than on the WD (Figure 2B), and the difference was related to a higher faecal content (727 + 49 mL vs. 452 + 21 mL, respectively; *p* < 0.001). To note, the difference in faecal content was consistent in all colonic compartments. The total colonic gas content was similar on the FMD and the WD diet (129 + 13 vs. 120 + 15, respectively; *p* = 0.307). However, the distribution of gas within the lumen was different. On the FMD diet, gas accumulated in the proximal colon (ascending plus transverse colon, 82 + 9 mL vs. 50 + 6 mL on the WD, *p* = 0.007) and the content was less in the distal colon (descending plus pelvic colon, 51 + 6 mL vs. 73 + 11 mL on the WD; *p* = 0.035).

### 3.7. Microbiome Metagenomic Analysis

A general taxonomic analysis was first performed to characterise microbial communities. In addition, the alpha diversity, measuring the variability of species within a sample, was determined. Mean values of the Chao1 index for WD and FMD intervention groups were similar (89.8 and 90.1, respectively). Other alpha diversity metrics were also calculated, including Shannon, Simpson and Inverse Simpson indices, showing no significant differences but slightly higher dispersion for FMD samples (Supplementary Figure S1). 

Principal coordinates analysis (PCoA) of complete microbial taxa in samples obtained after WD or FMD showed no major differences in the global taxonomic profiles of participants (Supplementary Figure S2). However, some samples were grouped. This fact could be attributed to individual factors other than the intervention and highlight the importance of inter-individual variability. A complementary canonical correspondence analysis (CCA) of samples at the genus level was also performed (Supplementary Figure S3). Similarly, differences in sample distribution could not be attributed to the type of diet and corroborate the effect of inter-individual variability. It should be noted that several samples were differentiated depending on the abundance of *Roseburia*, *Prevotella* and *Bacteroides* genus, regardless of dietary intervention.

**Figure 3:**
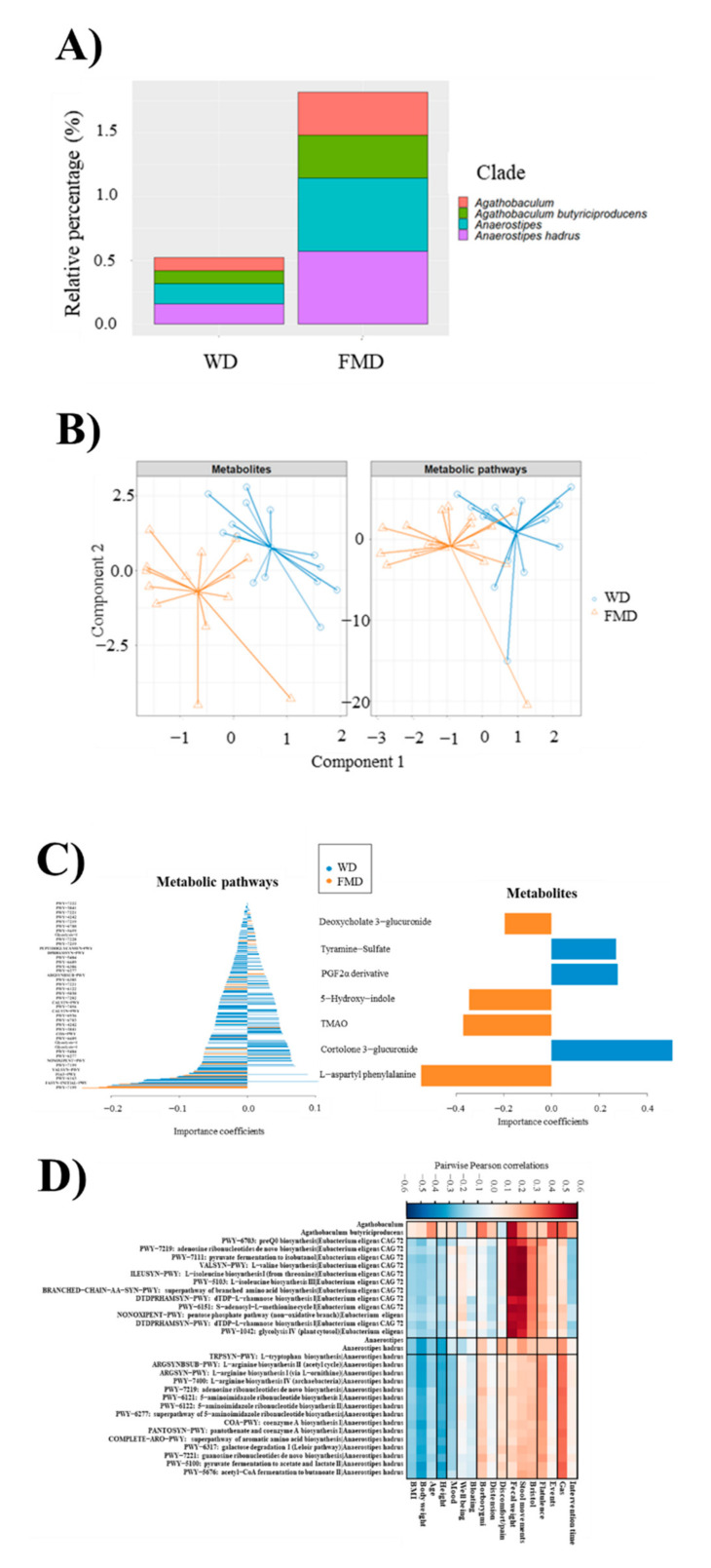
(**A**) Taxonomic clades more abundant in samples after Fibre-Enriched Mediterranean-Type Diet (FMD) intervention. Agathobaculum and Anaerostipes genus as well as Agathobaculum butyriciproducens and Anaerostipes hadrus species showed significantly higher (*p* < 0.05) abundances, assessed by DESeq2 differential abundance testing, in FMD samples. (**B**) Differential profiles obtained for Western-Type Diet (WD) and FMD samples through multi-omic data integration of metabolic profiles and metabolic pathways determined by shotgun sequencing using DIABLO biomarker discovery pipeline. (**C**) Variable importance coefficients obtained in DIABLO pipeline for metabolites and microbial pathways in WD and FMD interventions. TMAO: Trimethylamine N-oxide. PWY-7222: guanosine deoxyribonucleotides de novo biosynthesis. PWY-3841: folate transformations. PWY-7221: guanosine ribonucleotides de novo biosynthesis. PWY-4242: pantothenate and coenzyme A biosynthesis. PWY-7219: adenosine ribonucleotides de novo biosynthesis. PWY-6700: queuosine biosynthesis. PWY-5659: GDP-mannose biosynthesis. Glycolysis-1: glycolysis (from glucose 6-phosphate). PWY-7228: superpathway of guanosine nucleotides de novo biosynthesis. PEPTIDOGLYCANSYN-PWY: peptidoglycan biosynthesis. DTDPRHAMSYN-PWY: dTDP-L-rhamnose biosynthesis. PWY-5484: glycolysis II (from fructose 6-phosphate). PWY-6609: adenine and adenosine salvage. PWY-6386: UDP-N-acetylmuramoyl-pentapeptide biosynthesis. PWY-6277: superpathway of 5-aminoimidazole ribonucleotide biosynthesis. ARGSYNBSUB-PWY: L-arginine biosynthesis. PWY-6385: peptidoglycan biosynthesis. PWY-6122: 5-aminoimidazole ribonucleotide biosynthesis. PWY-5030: L-histidine degradation. PWY-7282: 4-amino-2-methyl-5-phosphomethylpyrimidine biosynthesis. CALVIN-PWY: Calvin–Benson–Bassham cycle. PWY-7456: mannan degradation. PWY-6936: seleno-amino acid biosynthesis. PWY-6703: preQ0 biosynthesis. COA-PWY: coenzyme A biosynthesis. NONOXIPENT-PWY: pentose phosphate pathway. PWY-7199: pyrimidine deoxyribonucleosides salvage. VALSYN-PWY: L-valine biosynthesis. P163-PWY: L-lysine fermentation to acetate and butanoate. PWY-6163: chorismate biosynthesis from 3-dehydroquinate. FASYN-INITIAL-PWY: superpathway of fatty acid biosynthesis initiation. The following codes include multiple metabolic pathways for different microorganisms: Calvin-PWY (*Bacteroides caccae* and *B. uniformis*), Glycolysis-1 (*B. uniformis, B. vulgatus* and *B. caccae*), PWY-3841 (*B. uniformis* and *B. vulgatus*), PWY-4242 (*B. uniformis* and *B. vulgatus*), PWY-5484 (*B. uniformis* and *B. vulgatus*), PWY-6277 (*B. uniformis* and *B. vulgatus*), PWY-6609 (*B. vulgatus* and *B. caccae*), PWY-7199 (*B. uniformis* and *B. vulgatus*), PWY-7219 (*B. uniformis* and *Anaerostipes hadrus*), PWY-7221 (*B. uniformis* and *A. hadrus*). (**D**) Hierarchical all-against-all association testing (HAllA) describing the effect of taxonomic clades and metabolic pathways more abundant after FMD on several clinical parameters, considering pairwise Pearson correlation coefficients adjusted by false discovery rate (FDR < 0.25). Red and blue cells indicate positive and negative correlations, respectively. Colour intensity is in proportion to magnitude.

Taxonomic analysis at the species level was performed. Cladogram calculated from phylogenetic distances reveals the presence of *Anaerostipes hadrus* (Supplementary Figure S4), which showed high abundances after FMD (Figure 3A). In fact, *Agathobaculum* and *Anaerostipes* genus, as well as *Agathobaculum butyriciproducens* and *Anaerostipes hadrus* species, showed significantly higher (*p* < 0.05) abundances, assessed by DESeq2 differential abundance testing, after FMD intervention (Figure 3A). These results indicate that FMD modulated the growth of specific taxa, showing no major effects on the core microbiota.

No statistically significant differences were found between gene families present in samples from WD and FMD interventions. Moreover, no carry-over effect was observed when analysing data according to randomisation order (Supplementary Figure S5). However, beta diversity values calculated from the expression values of metabolic pathways were higher in FMD than in WD samples (Supplementary Figure S6). A total of 27 metabolic pathways showed significantly higher expression after FMD intervention.

### 3.8. Metabolomic Analysis

Multivariate analyses revealed a differential pattern in the metabolome of urine samples from individuals who consumed FMD compared to WD (Figure 3B). A total of 309 and 279 metabolites were significantly increased (≥2-fold) after consuming the FMD and WD, respectively (Supplementary Figure S7). However, many of these significant metabolites did not yield an accurate mass-based putative identification when searched against several databases and online libraries. For this reason, we first selected all the metabolites with a predictive score above 95%. Secondly, from this initial list of metabolites, despite the coincidence of the exact molecular formula, we removed all the tentative identifications provided by the software that were somewhat irrelevant or non-related with the dietary intervention (i.e., common dietary-derived molecules such as caffeine and others, atmospheric pollutants, pharmaceutical drugs, pesticides, etc.). Following these criteria, 11 metabolic entities were selected for further validation (six of them significantly increased after FMD, and the other five, significantly increased after WD) (Supplementary Table S1). 

After normalising diuresis and comparing with available standards, the following metabolites were confirmed to be significantly increased after FMD: deoxycholate glucuronide (2.1-fold), 5-hydroxyindole (2-fold), L-aspartyl-L-phenylalanine (2.4-fold) and TMAO (1.5-fold). The metabolites propionyl-L-carnitine (2.2.-fold), cortolone 3-glucuronide (2-fold), *p*-tyramine 3-sulphate (2.4-fold) and 18-acetoxy-PGF2α-11-acetate methyl ester (2-fold) were confirmed to be significantly increased after consuming the WD (Supplementary Table S1). No standards were available for the conjugated metabolites (mainly glucuronide or sulphate derivatives). In this case, the confirmation was indirectly inferred after detecting the free form (commercially available) upon glucuronidase/sulfatase treatment. This was the case of deoxycholic acid, cortolone, *p*-tyramine and PGF2α. In the entity with exact mass 467.2641, two possible metabolites were tentatively identified by the libraries, i.e., 18-acetoxy-PGF2α-11-acetate methyl ester and 3-α-androstanediol glucuronide. After enzymatic treatment, the prostaglandin F2α (PGF2α) was confirmed after comparing it with its authentic standard. However, the tentatively identified precursor was not either glucuronidated or sulphated. In this case, we confirmed that the assay conditions used in the enzymatic treatment (incubation time, pH and temperature) were enough to release the unconjugated PGF2α. Therefore, it is reasonable to tentatively accept the metabolite 18-acetoxy-PGF2α-11-acetate methyl ester, taking into account the predictive score and the confirmation of the released PGF2α. However, we acknowledge we could not unequivocally assign the exact mass 467.2641 to 18-acetoxy-PGF2α-11-acetate methyl ester. No confirmation was possible for the exact mass 242.2122 (tentatively: *N*-phenylacetic aspartic acid, carboxy acetyl D-phenylalanine, or *N*-feruloylglycine) due to the lack of standards. Similarly, the entity with exact mass 242.2122 that increased 16-fold after consuming WD, and tentatively identified as sphingosine, as well as the entity 381.0800 that increased 2.7-fold after FMD, tentatively 3,4-dihydroxyphenyl propionic acid glucuronide, were not confirmed after comparison with their corresponding standards.

### 3.9. Multi-Omic Analysis

To correlate all data generated and better characterise different metabolic and metagenomic profiles obtained after WD and FMD, multi-omic methods were employed. Specifically, metabolite levels and metabolic pathways determined by shotgun sequencing were integrated using the DIABLO biomarker discovery pipeline. This modelling technique revealed different profiles for most WD and FMD samples (Figure 3B). It should be considered that data integration methods allow determining complex patterns in biological samples that could not be inferred using conventional techniques such as PCA, as previously discussed (Supplementary Figure S8). Variable importance coefficients were calculated for each metabolic pathway (Figure 3C) and metabolite (Figure 3D) to interpret these profiles. As a consequence, it was found that metabolic profiles were characterised by the expression of the following pathways in FMD samples: pentose phosphate pathway, pyrimidine deoxyribonucleosides salvage, L-valine biosynthesis, L-lysine fermentation to acetate and butanoate, chorismate biosynthesis from 3-dehydroquinate and superpathway of fatty acid biosynthesis initiation (Figure 3C). In addition, cortolone 3-glucuronide and L-aspartyl phenylalanine were the most characteristic compounds from WD and FMD metabolite analysis, respectively, showing significantly (*p* < 0.05) higher abundances after these interventions.

HAllA testing was performed to investigate potential associations between microbial metabolism and clinical symptoms. This model described the effect of the most relevant taxonomic clades and metabolic pathways found after FMD on clinical parameters, considering pairwise Pearson correlation coefficients adjusted by false discovery rate (FDR < 0.25) (Figure 3D). A positive association was found between *Agathobaculum butyriciproducens* and faecal weight. A similar association was determined for several pathways from *Eubacterium eligens* CAG 72, involved in carbohydrate and amino acid metabolism, and faecal weight and stool movements. Finally, a positive influence on gas production was observed for *Anaerostipes hadrus* and different pathways involved in ribonucleotide, carbohydrate and amino acid metabolism.

### 3.10. Robust and Non-Robust Microbiotas

**Figure 4:**
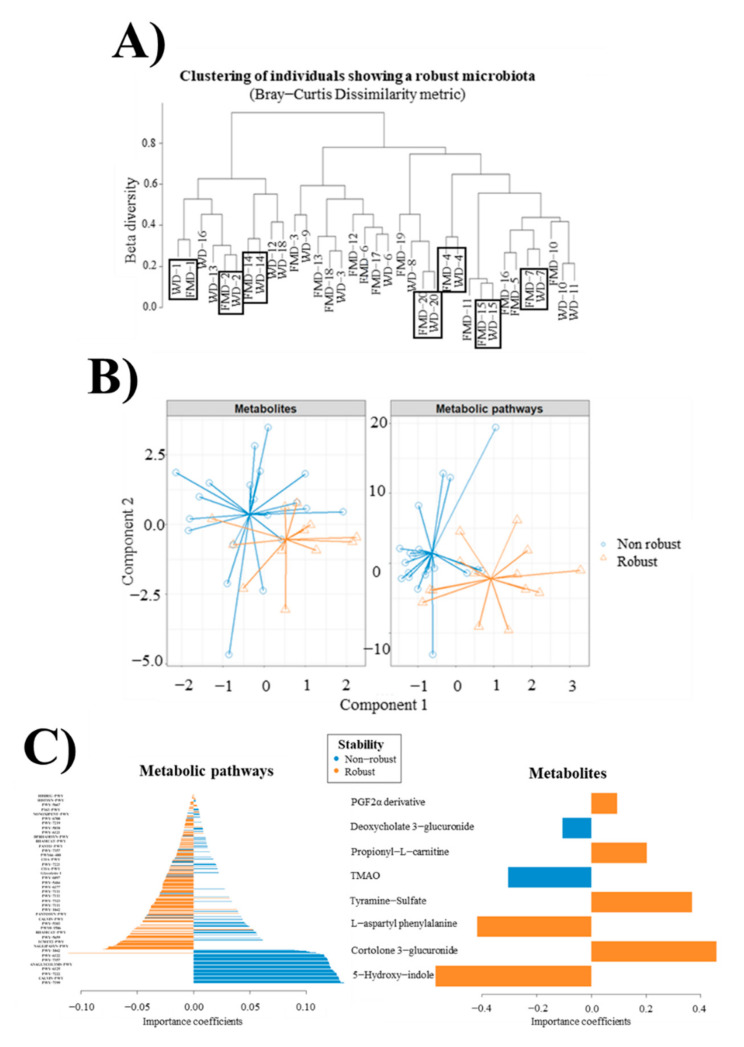

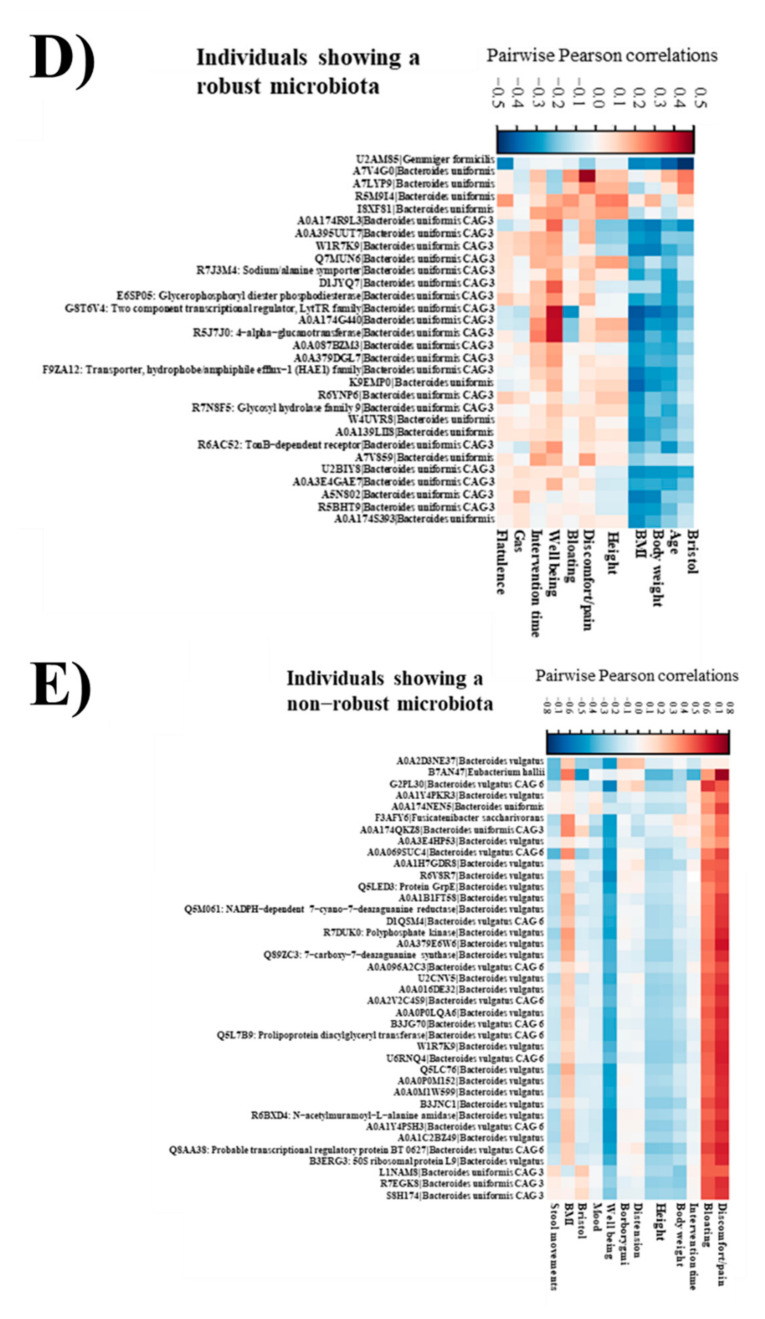
(**A**) Clustering of metagenomic samples of metabolic pathway abundances using. Bray–Curtis dissimilarity method was used to determine which participants were grouped. Several samples from the same participant corresponding to Western-Type Diet (WD) and Fibre-Enriched Mediterranean-Type Diet (FMD) interventions were discriminated from the rest of the individuals, highlighting a robust microbiota was not significantly affected by dietary interventions. (**B**) Differential profiles for robust and non-robust microbiotas through multi-omic data integration of metabolic profiles and metabolic pathways determined by shotgun sequencing using the DIABLO biomarker discovery pipeline. (**C**) Variable importance coefficients obtained in DIABLO pipeline for metabolites and metabolic pathways in robust and non-robust microbiotas. TMAO: Trimethylamine N-oxide. HISTSYN-PWY: L-histidine biosynthesis. HISDEG-PWY: L-histidine degradation. PWY-5667: CDP-diacylglycerol biosynthesis. P163-PWY: L-lysine fermentation to acetate and butanoate. NONOXIPENT-PWY: pentose phosphate pathway. PWY-6700: queuosine biosynthesis. PWY-7219: adenosine ribonucleotides de novo biosynthesis. PWY-5030: L-histidine degradation. PWY-6121: 5-aminoimidazole ribonucleotide biosynthesis. DTDPRHAMSYN-PWY: dTDP-L-rhamnose biosynthesis. RHAMCAT-PWY: L-rhamnose degradation. PANTO-PWY: phosphopantothenate biosynthesis. PWY-7357: thiamin formation from pyrithiamine and oxythiamine. PWY66–400: glycolysis VI. COA-PWY: coenzyme A biosynthesis. PWY-7221: guanosine ribonucleotides de novo biosynthesis. Glycolysis-1: glycolysis (from glucose 6-phosphate). PWY-6897: thiamin salvage. PWY-5484: glycolysis (from fructose 6-phosphate). PWY 6277: superpathway of 5-aminoimidazole ribonucleotide biosynthesis. PWY-7111: pyruvate fermentation to isobutanol. PWY-7323: superpathway of GDP-mannose-derived O-antigen building blocks biosynthesis. PWY-1042: glycolysis. PANTOSYN-PWY: pantothenate and coenzyme A biosynthesis. CALVIN-PWY: Calvin–Benson–Bassham cycle. PWY-5103: L-isoleucine biosynthesis. PWY0-1586: peptidoglycan maturation. PWY-5659: GDP-mannose biosynthesis. 1CMET2-PWY: N10-formyl-tetrahydrofolate biosynthesis. NAGLIPASYN-PWY: lipid IVA biosynthesis. PWY-6122: 5-aminoimidazole ribonucleotide biosynthesis. ANAGLYCOLYSIS-PWY: glycolysis (from glucose). PWY-6125: superpathway of guanosine nucleotides de novo biosynthesis. PWY-7222: guanosine deoxyribonucleotides de novo biosynthesis. PWY-7199: pyrimidine deoxyribonucleosides salvage. The following codes include multiple metabolic pathways for different microorganisms: CALVIN-PWY (*Bacteroides caccae* and *B. uniformis*), RHAMCAT-PWY (*B. vulgatus* and *B. caccae*), PWY-1042 (*B. uniformis* and *B. vulgatus*), PWY-7357 (*B. uniformis* and *B. caccae*), PWY-7111 (*B. uniformis*, *B. vulgatus* and *B. caccae*). Hierarchical all-against-all association testing (HAllA) describing the effect of microbial gene families from a robust (**D**) and non-robust (**E**) microbiota on several clinical parameters, considering pairwise Pearson correlation coefficients adjusted by false discovery rate (FDR < 0.25). Red and blue cells indicate positive and negative correlations, respectively. Colour intensity is in proportion to magnitude.

Dietary intervention exerted a relevant effect on specific metabolic pathways, although changes were less evident in some participants. The characteristics of those metagenomes that remained stable were investigated. For this purpose, metagenomic samples were clustered using the Bray–Curtis dissimilarity metric of metabolic pathway abundances to determine which participants were grouped. In some cases, samples from the same participant corresponding to WD and FMD interventions were discriminated from the rest of the individuals, highlighting a robust microbiota that was scarcely affected by dietary interventions. As shown in Figure 4A, the dietary intervention exerted less impact in participants 1, 2, 4, 7, 14, 15 and 20 than in the remainder.

PCA of participants’ clinical parameters, showing a robust and non-robust microbiota, indicated no significant differences in the complete set of clinical symptoms according to the stability of the microbiota (Supplementary Figure S9). The absence of global clinical patterns that could be attributed to the stability of the microbiota is also observed when only WD and FMD groups are considered (Supplementary Figures S10 and S11). This fact can be attributed to the lack of significant differences between participants and the presence of high inter-individual variability, as previously explained (Supplementary Figure S8). No relevant relationships between the stability of the microbiota and clinical symptoms could be inferred. However, participants showing higher body height and weight also showed a significantly higher (*p* < 0.05) stability of the microbiota after WD and FMD interventions (Supplementary Figure S12). The increase in intracolonic faecal content during the FMD diet tended to be lower in robust (146 ± 69 mL) than in non-robust individuals (307 ± 73 mL, n.s.). Change in anal gas evacuations during FMD was also less prominent in robust (1 ± 4) than in non-robust individuals (10 ± 3, n.s.). Beta-diversity indicators calculated on gene families and metabolic pathways were slightly higher in robust microbiotas (Supplementary Figures S13 and S14).

DESeq2 differential abundance, testing for sequencing data, was then applied to elucidate characteristic metabolic patterns of robust and non-robust microbiotas. Statistically significant differences (*p* < 0.05) between 8319 gene families were associated with specific microbial species present in individuals, showing a robust and non-robust microbiota (Supplementary Table S2). A total of 7628 gene families showed significantly higher abundances in robust microbiotas. Most of these gene families corresponded to *Bacteroides* genus, including *Bacteroides uniformis* and *Bacteroides uniformis* CAG 3 strain, as well as *Bacteroides vulgatus* and *Bacteroides vulgatus CAG 6*. Different gene families from *Bacteroides caccae, Bacteroides ovatus* and *Bacteroides xylanisolvens* were also characteristic of this microbiota type. Distinctive gene families from robust microbiotas also included gene families from *Parabacteroides distasonis*, *Parabacteroides merdae* and *Ruminococcus bromii*. On the other hand, non-robust microbiotas were also characterised by a lower number of gene families from these species, as well as *Gemmiger formicilis* and *Bacteroides thetaiotaomicron*. Although most gene families present in robust and non-robust microbiotas belonged to the same species, the biological functions of these genes were different. Up to 1322 gene families with known associated functions were found in robust microbiotas, including peptidyl-prolyl cis-trans isomerases, ABC transporters, outer membrane receptors, transcriptional regulators, glycosidases, transposases and recombinases. In contrast, 134 genes with different associated functions were determined in non-robust microbiotas involving beta-lactamases, kinases, L-aspartate oxidases, oligopeptide transporter and sodium/alanine symporters.

Similarly, 250 statistically significant (*p* < 0.05) metabolic pathways, expressed in individuals showing a robust and non-robust microbiota, were found (Supplementary Table S3). Robust microbiotas showed 164 distinct metabolic pathways mainly from *Bacteroides* species in agreement with gene family data, as well as *Flavonifractor plautii* and *Prevotella copri*. Non-robust microbiotas were characterised by 86 metabolic pathways from *Bacteroides*, *Gemmiger formicilis*, *Parabacteroides distasonis*, *Blautia obeum* and *Blautia wexlerae*. Among the most represented pathways of robust microbiotas, the Calvin–Benson–Bassham cycle, pyruvate fermentation to isobutanol and GDP-mannose biosynthesis were not highly represented in non-robust microbiotas. On the other hand, dTDP-L-rhamnose biosynthesis and phosphopantothenate biosynthesis were characteristic of non-robust microbiotas.

A multi-omic integration model was applied to metabolite and metabolic pathways data, obtaining two different profiles for robust and non-robust microbiotas (Figure 4B). These profiles were mainly defined by a series of metabolic pathways that were more characteristic of non-robust microbiotas, including pantothenate and coenzyme A biosynthesis, L-isoleucine biosynthesis, peptidoglycan maturation, N10-formyl-tetrahydrofolate biosynthesis, lipid IVA biosynthesis, superpathway of guanosine nucleotides de novo biosynthesis, guanosine deoxyribonucleotides de novo biosynthesis and pyrimidine deoxyribonucleosides salvage (Figure 4C). Concerning the metabolite profiles, the production of cortolone 3-glucuronide and 5-hydroxy-indole was more accentuated in robust microbiotas (Figure 4D).

HAllA model revealed the negative association between several gene families from *Bacteroides uniformis* and *Bacteroides uniformis* CAG strain and Bristol index and body weight in robust microbiotas (Figure 4E). Some of these gene families also contributed to improving well-being, including 4-alpha-glucanotransferase and a transcriptional regulator from LytTR family. On the contrary, several gene families from these microorganisms were strongly associated with discomfort and abdominal pain in non-robust microbiotas (Figure 4E). These results may indicate that participants showing non-robust microbiotas could be more prone to suffer abdominal pain, probably due to those changes in metagenomes induced by diet.

## 4. Discussion 

Our study shows that diet influences the content of biomass in the colon, faecal output, microbiota metabolic activity, gas production by fermentative processes and perception of digestive sensations, with some effects on microbiota composition. 

The healthy participants in this study tolerated well both diet plans. The FMD with higher content in residues increased colonic biomass, as evidenced by luminal content measurements in the unprepared colon using MRI. The turn-over of colonic biomass also increased, as shown by the increase in faecal output. Higher residues in the diet increased intestinal gas production with increased anal gas evacuation. Remarkably, intestinal gas evacuation measurements can be used as a non-invasive surrogate marker of microbiota metabolic activity. Interestingly, anal gas evacuation matched the production rate and prevented intraluminal pooling of gas. Previous studies have shown that intestinal gas is under tight homeostatic control [34]. Still, despite the stability of total gas content, the distribution of intraluminal gas changed, probably due to regional changes in microbiota activity with higher fermentation rates in the proximal colon on the high-residue diet. Changes in the volume of biomass and gas metabolism were associated with digestive sensations, particularly abdominal bloating and distension, without impacting digestive well-being and mood. Previous studies showed that ingestion of a comfort meal induces homeostatic sensations (satiety, fullness) with a positive hedonic dimension involving digestive well-being and mood, fat being one of the meal components related to the hedonic response. Contrary to our hypothesis, as compared to the WD, fat restriction on the FMD did not increase the rewarding response to the fat-rich comfort meal.

Taxonomic profiles of microbial communities in faecal samples were similar at the end of WD or FMD, suggesting little influence of the diet on the core members of the gut microbiota of each individual. Interestingly, the abundance of butyrate producers such as *A. butyriciproducens* and *A. hadrus* was significantly higher after FMD than WD. This finding is functionally relevant considering the fundamental role of butyrate producers in colonic homeostasis [35]. Associations were found between abundance of butyrate producers, faecal weight and gas volume evacuated after the probe meal.

In contrast, the impact of diet on gut microbial metabolism was remarkable, and the two diet plans shifted microbial metabolism towards clearly distinctive patterns. Microbial metabolic pathways in faecal samples and urinary metabolite profiles were significantly different at the end of the intervention periods. First, the beta-diversity of microbial metabolic pathways was higher after FMD than WD, suggesting that several metabolic pathways were only detectable after FMD. Furthermore, a total of 27 metabolic pathways showed significantly higher expression after FMD intervention. Thus, our findings suggest that dietary substrates reaching the colon have an important impact on microbial metabolic functions without notable changes in core community members. The metabolic potential of the community members rapidly adapts to changes in substrate availability. In healthy subjects, the hidden metabolic potential of the microbiome can be rapidly recovered with appropriate foods. However, this might not be the case in individuals with long-term dietary restrictions.

Increased urinary compounds after WD included carnitine and tyramine metabolites, which might be related to the ingestion of meat and cheese, and cortisol and prostaglandin metabolites, reflecting the enhanced systemic inflammatory background that has been reported after high-fat meals [36,37]. After FMD, increased urinary levels of TMAO might be explained by the ingestion of some plant foods rich in choline, such as legumes, on the days before urine sampling [38]. The excretion of deoxycholate-glucuronide in urine increased after the FMD, which certainly contained less fat and cholesterol than the WD. People on Mediterranean diet tend to have lower blood cholesterol levels than people on WD, and we speculate that enhanced microbial glucuronidation of bile acids would be an additional mechanism to explain lower blood cholesterol in subjects on FMD. Glucuronidation is a major detoxification pathway for bile acids [39], since the resulting glucuronide products are soluble and more easily excreted in urine than the initial molecules.

Of interest, some individuals harboured gut metagenomes that were less influenced by the dietary plans, and variation in microbial pathways was less evident than in the other participants. Based on this stratification rule, we compared robust versus non-robust microbiotas. Robust microbiotas had higher diversity than the others and were enriched in metabolic pathways associated with species from *Bacteroides, Parabacteroides* and *Ruminococcus* genera. Urinary metabolite profiles also differed between robust and non-robust microbiotas. Finally, individuals harbouring a robust microbiota experienced less change in faecal contents and anal gas evacuations during FMD. However, we cannot ascertain to what extent the stability of the microbiota was related to preconditioning factors, because previous diet, lifestyle and physical activity, which may be determinant of microbiome remodelling, were not checked.

We wish to acknowledge that this proof-of-concept study included a relatively small sample; to enhance the homogeneity of the study group, only healthy men were studied, and sex could affect the results. Nevertheless, the outcomes suggest that the method is scientifically valid and could also be applied to specific categories of subjects with symptoms, who may respond differently from healthy subjects. 

## 5. Conclusions

The FMD, a diet rich in fruits, vegetables and legumes, increased the volume of colonic biomass by 60% and the relative abundance of specific butyrate-producing taxa, pointing out a remarkable expansion of the microbial load of fermentative species within the colon [40], without major changes in the core microbiota composition. This was also manifested by an increase in intestinal gas production, higher abundance of a number of microbial metabolic pathways and a distinct urinary pattern of microbial metabolites after the plant-rich diet, which was well tolerated in healthy individuals. We conclude that dietary variations may induce subtle changes in the relative abundance of microbial taxa but have a strong influence on microbial metabolism.

## Data Availability

Metagenomic sequencing data files are publicly available at the Sequence Read Archive (SRA) of the National Center for Biotechnology Information (NCBI) including raw paired-end reads in FASTQ format. Data were deposited under provisional code SUB10001498.

## Supplementary Materials

The following are available online at https://www.mdpi.com/article/10.3390/nu13082638/s1, Summary of WD and FMD diets, Supplementary Material, Figure S1: Canonical correspondence analysis (CCA) at genus level of samples obtained after Western-type Diet (WD) or Fiber-enriched Mediterranean-type Diet (FMD) interventions. CA: correspondence analysis. Some samples were grouped similar to principal coordinate analysis (PCoA) (See Supplementary Material Figure S2). Differences in sample distribution might be due to additional factors other than diet, highlighting the importance of inter-individual variability. The percentage of variance explained by each CA is indicated in the axis, Figure S2: Cladogram calculated from phylogenetic distances of microbial species found in metagenomic samples from participants. Among species analysed, *Anaerostipes hadrus* showed high abundances after Fiber-enriched Mediterranean-type Diet (FMD) (for further details, see Figure 1), Figure S3: Carry-over effects observed for propionyl-L-carnitine (A) and Cortolone 3-glucuronide (B) levels in samples obtained after Western-type Diet (WD) or Fiber-enriched Mediterranean-type Diet (FMD) intervention at 1st or 2nd period, Figure S4: Beta-diversity analysis of metabolic pathways determined in samples obtained after Western-type Diet (WD) or Fiber-enriched Mediterranean-type Diet (FMD) interventions. The Bray-Curtis method was selected for the calculation. A total of 27 metabolic pathways showed significantly higher expresión after FMD intervention, Figure S5: Principal coordinates analysis (PCoA) of taxa present in samples obtained after Western-type Diet (WD) or Fiber-enriched Mediterranean-type Diet (FMD) interventions. No significant differences can be observed in the global taxonomic profiles of participants according to the diet. PC: principal coordinate. The percentage of variance explained by each PC is indicated in the axis, Figure S6: Venn diagrams (A, B) and three-dimension Principal Component Analyses (PCA) (C, D) showing those metabolites significantly increased (≥2-fold) after WD (orange colour) and after FMD (blue colour) in UPLC-QTOF-MS positive (A, C) and negative (B, D) detection modes, Figure S7: Beta-diversity analysis of gene families (A, B) and metabolic pathways (C, D) determined in samples from participants showing a robust and non-robust microbiota after Western-type Diet (WD) or Fibre-enriched Mediterranean-type Diet (FMD) interventions (for further details, see Figure 2). The Bray-Curtis method was selected for the calculation. Beta-diversity values were slightly higher in robust microbiotas that did not suffer any relevant change after WD (A, C), Figure S8: Comparison of different alpha-diversity indicators (Shannon, Simpson and Inverse Simpson) of the relative abundance of taxa determined after Western-type Diet (WD) or Fiber-enriched Mediterranean-type Diet (FMD) interventions, Figure S9: Principal components analysis (PCA) of clinical parameters of participants after Western-type Diet (WD) or Fiber-enriched Mediterranean-type Diet (FMD) interventions. As it can be seen, no relevant differences in clinical symptoms can be observed according to the type of diets administered. Dim: dimension (principal component), BMI: Body mass index. The percentage of variance explained by each Dim is indicated in the axis, Figure S10: Principal components analysis (PCA) of clinical parameters of participants showing a robust and non-robust microbiota (for further details, see Figure 2). No relevant differences in clinical symptoms can be observed according to the stability of the microbiota. Dim: dimension (principal component), BMI: Body mass index. The percentage of variance explained by each Dim is indicated in the axis, Figure S11: Principal components analysis (PCA) of clinical parameters of participants showing a robust and non-robust microbiota after Western-type Diet (WD) intervention (for further details, see Figure 2). No relevant differences in clinical symptoms can be observed according to the stability of the microbiota. Dim: dimension (principal component), BMI: Body mass index. The percentage of variance explained by each Dim is indicated in the axis, Figure S12: Principal components analysis (PCA) of clinical parameters of participants showing a robust and non-robust microbiota after Mediterranean-type Diet (FMD) intervention (for further details, see Figure 2). No relevant differences in clinical symptoms can be observed according to the stability of the microbiota. Dim: dimension (principal component), BMI: Body mass index. The percentage of variance explained by each Dim is indicated in the axis, Figure S13: Influence of height and body weight on the stability of the microbiota (for further details, see Figure 2). Participants showing higher height and body weight also showed higher microbiota stability after Western-type Diet (WD) and Fiber-enriched Mediterranean-type Diet (FMD) interventions, Figure S14: Beta-diversity analysis of gene families (A) metabolic pathways (B) determined in samples from participants showing a robust and non-robust microbiota (for further details, see Figure 2). The Bray-Curtis method was selected for the calculation. Beta-diversity values were slightly higher in robust microbiotas that did not suffer any relevant change after Western-type Diet (WD) and Fiber-enriched Mediterranean-type Diet (FMD) interventions, Table S1: Metabolites significantly different in urine after consumption of FMD and WD, Table S2: Characteristic microbial gene families present in individuals showing a robust and non-robust microbiota (see Figure 2), Table S3: Distinctive microbial metabolic pathways expressed in individuals showing a robust and non-robust microbiota (see Figure 2).
