# Supplementary material for: Differential Effects of Western and Mediterranean-Type Diets on Gut Microbiota: A Metagenomics and Metabolomics Approach
Source: Nutrients. 2021 Jul 30;13(8):2638. doi: 10.3390/nu13082638 (PMC8400818; doi:10.3390/nu13082638)
Supplement: Supplementary file 1 [file nutrients-13-02638-s001.zip › nutrients-1289360-supplementary.pdf]

Summary of WD and FMD diets

Western-type diet (WD)									
	Calories (Kcal)	Total fat (g)	Saturated fat (g)	Carbohydrate (g)	Protein (g)	Fiber (g)	G%	CHO%	P%
Day 1	2380,9	135,7	53,2	156,7	122,9	4,4	51	26	21
Day 2	2368,1	130,1	43,6	155,8	135,7	3,1	49	26	23
Day 3	2373,1	130,1	47,1	168,0	122,5	4,8	49	28	21
Day 4	2366,9	134,5	48,2	162,6	116,5	7,4	51	27	20
Day 5	2319,7	126,9	50,6	168,9	123,2	4,9	49	29	21
Day 6	2250,7	127,7	53,9	160,1	106,6	4,1	51	28	19
Day 7	2387,7	146,9	58,0	138,5	120,3	4,0	55	23	20
Media	2349,6	133,1	50,7	158,7	121,1	4,7	51	27	21

Fibre-enriched Mediterranean diet (FMD)									
	Calories (Kcal)	Total fat (g)	Saturated fat (g)	Carbohydrate (g)	Protein (g)	Fiber (g)	G%	CHO%	P%
Day 1	2339,7	51,6	6,6	364,5	83,3	56,3	20	62	14
Day 2	2319,0	47,9	3,4	359,1	96,7	58,8	19	62	17
Day 3	2336,9	55,5	5,1	329,1	92,5	53,8	21	56	16
Day 4	2357,1	49,6	4,3	366,7	93,7	49,9	19	62	16
Day 5	2325,9	54,6	5,4	363,0	81,8	50,6	21	62	14
Day 6	2306,0	45,2	3,1	366,9	95,4	55,3	18	64	17
Day 7	2382,7	43,2	3,8	391,4	91,3	54,4	16	66	15
Media	2338,2	49,6	4,5	362,9	90,7	54,2	19	62	16

Days 1 and 8 FMD

**Day 1**	**gr**	**Kcal**	**Fat**	**Saturated fat**	**Carbohydrates**	**Protein**	**Fiber**
**Breakfast**							
Low-fat milk	200	92,0	3,2	2,2	9,6	6,2	0,0
Bacon	54	188,5	17,1	6,9	0,5	8,0	0,0
Egg	68	102,0	7,5	0,0	0,3	8,5	0,0
Crackets	30	143,4	5,7	0,6	20,1	2,5	0,5
Sugar	14	56,0	0,0	0,0	14,0	0,0	0,0
**Lunch**							
Bread	21	56,5	0,8	0,1	10,1	1,9	0,6
Bratwurst Sausage	200	399,4	28,1	11,0	2,4	33,6	0,0
Noodles	40	141,6	0,6	0,1	28,8	4,4	1,6
Butter	12	89,2	9,8	6,7	0,1	0,1	0,0
Choco cookie bar	16	85,3	4,7	2,9	9,7	1,0	0,2
**Snack**							
Milk bread	40	138,0	2,1	1,3	20,4	3,8	0,9
Ham	31,5	62,7	1,6	0,6	0,2	12,0	0,0
Butter	12	89,2	9,8	6,7	0,1	0,1	0,0
**Dinner**							
Bread	21	36,3	0,3	0,1	7,5	0,9	0,1
Potato omelette	200	316,0	17,8	2,8	28,0	8,8	2,9
Cheese	18	37,9	2,2	1,4	1,6	2,5	0,4
Pork chop	150	316,5	22,5	8,7	0,0	28,5	0,0
Chocolate	5,5	30,8	1,9	1,1	3,2	0,3	0,0
**Total**		**2381**	**136**	**53**	**157**	**123**	**7**
**Breakfast**		582	34	10	45	25	1
**Lunch**		772	44	21	51	41	2
**Snack**		290	34	13	58	28	4
**Dinner**		738	43	13	37	41	3

Days 2 and 9 FMD

Day 2	gr	Kcal	Fat	Saturated fat	Carbohydrates	Protein	Fiber
**Breakfast**							
Low-fat milk	200	92,0	3,2	2,2	9,6	6,2	0,0
Salami	60	277,2	25,2	7,8	3,06	9,6	0
Egg	68	102,7	7,5	0,0	0,3	8,5	0,0
Crackers	30	143,4	5,7	0,6	20,1	2,5	0,5
Sugar	14	56,0	0,0	0,0	14,0	0,0	0,0
**Lunch**							
Bread	21	56,5	0,8	0,1	10,1	1,9	0,6
Cannelloni with meat, bechamel and cheese	300	513	30,3	8,7	23,4	36	0,0
Snicker	18	86,6	4,1	1,4	11,0	1,5	0,0
**Snack**							
Milk bread	40	138,0	2,1	1,3	20,4	3,8	0,9
Iberian chorizo	63	286,2	22,6	9,1	0,6	20,0	0,0
Butter	12	89,2	9,8	6,7	0,1	0,1	0,0
**Dinner**							
Bread	21	56,5	0,8	0,1	10,1	1,9	0,6
Chicken	140	161,0	1,7	0,6	4,3	32,2	0,0
Cheese	18	37,9	2,2	1,4	1,6	2,5	0,4
Ham croquettes	120	241,2	12,0	2,4	24,0	8,9	0,0
Chocolate	5,5	30,8	1,9	1,1	3,2	0,3	0,0
**TOTAL**		**2368**	**130**	**44**	**156**	**136**	**3**
**Breakfast**		671	42	11	47	27	1
**Lunch**		656	31	9	33	38	1
**Snack**		513	35	17	21	24	1
**Dinner**		527	19	6	43	46	1

Days 3 and 10 FMD

**Day 3**	**gr**	**Kcal**	**Fat (g)**	**Saturated fat (g)**	**Carbohydrates (g)**	**Protein (g)**	**Fiber (g)**
**Breakfast**							
Low-fat milk	200	92	3,2	2,2	9,6	6,2	0
Ham	60	61,2	1,9	0,7	4,3	6,8	0,0
Egg	68	102,7	7,5	0,0	0,3	8,5	0,0
Crackers	30	143,4	5,7	0,6	20,1	2,5	0,5
Sugar	14	56	0	0	14	0	0,0
**Lunch**							
Bread	21	56,5	0,8	0,1	10,1	1,9	0,6
Beef	150	165,0	4,5	0,0	0,0	31,4	0,0
Potato omelette	200	316	17,8	2,8	28	8,8	2,88
Macaroon	20	80,2	2,6	0,56	12	1,9	0,58
**Snack**							
Milk bread	40	138,0	2,1	1,3	20,4	3,8	0,9
Salami	60	277,2	25,2	7,8	3,1	9,6	0,0
Butter	12	89,2	9,8	6,7	0,1	0,1	0,0
**Dinner**							
Bread	21	56,5	0,8	0,1	10,1	1,9	0,6
Bratwurst sausage	200	399,4	28,1	11,0	2,4	33,6	0,0
Noodles	40	141,6	0,6	0,1	28,8	4,4	1,6
Butter	12	167,5	17,5	12,0	1,6	1,0	0,0
Chocolate	5,5	30,8	1,9	1,1	3,2	0,3	0,0
**Total**		**2373**	**130**	**47**	**168**	**122**	**8**
**Breakfast**		455	18	3	34	24	1
**Lunch**		618	26	3	50	44	4
**Snack**		504	37	16	24	13	1
**Dinner**		796	49	24	46	41	2

Days 4 and 11 FMD

Day 4	gr	Kcal	Fat (g)	Saturated fat (g)	Carbohydrates (g)	Protein (g)	Fiber (g)
**Breakfast**							
Low-fat milk	200	92,0	3,2	2,2	9,6	6,2	0,0
Bacon	54	188,5	17,1	6,9	0,5	8,0	0,0
Egg	68	102,0	7,5	0,0	0,3	8,5	0,0
Crackers	30	143,4	5,7	0,6	20,1	2,5	0,5
Sugar	14	56	0	0	14	0	0
**Lunch**							
Bread	21	56,5	0,8	0,1	10,1	1,9	0,6
Pork chop	150	316,5	22,5	8,7	0,0	28,5	0,0
Cheese	18	37,9	2,2	1,4	1,6	2,5	0,4
Russian salad	125	274,0	22,5	3,5	10,0	7,1	2,3
Snicker	18	86,6	4,1	1,4	11,0	1,5	0,2
**Snack**							
Milk bread	40	138,0	2,1	1,3	20,4	3,8	0,9
Iberian chorizo	63	286,2	22,6	9,1	0,6	20,0	0,0
Butter	12	89,2	9,8	6,7	0,1	0,1	0,0
**Dinner**							
Bread	21	56,5	0,8	0,1	10,1	1,9	0,6
Bolognese lasagna	300	375	9,3	3,6	49,5	21,3	3,9
Cheese	18	37,9	2,2	1,4	1,6	2,5	0,4
Chocolate	5,5	30,8	1,9	1,1	3,2	0,3	0,0
**Total**		**2367**	**135**	**48**	**163**	**117**	**10**
**Breakfast**		582	34	10	45	25	1
**Lunch**		771	48	14	22	40	3
**Snack**		513	35	17	21	24	1
**Dinner**		500	14	6	64	26	5

Days 5 and 12 FMD

**Day 5**	**gr**	**Kcal**	**Fat (g)**	**Saturated fat (g)**	**Carbohydrates (g)**	**Protein (g)**	**Fiber (g)**
**Breakfast**							
Low-fat milk	200	92,0	4,0	2,8	12,0	7,8	0,0
Salami	60	277,2	25,2	7,8	3,1	9,6	0,0
Egg	68	102,7	7,5	0,0	0,3	8,5	0,0
Crackers	30	143,4	5,7	0,6	20,1	2,5	0,5
Sugar	14	56	0	0	14	0	0
**Lunch**							
Bread	21	56,5	0,8	0,1	10,1	1,9	0,6
Pork Burguer	180	381,6	28,8	10,1	5,4	25,2	0,0
Cheese	18	37,9	2,2	1,4	1,6	2,5	0,4
Ham croquettes	120	241,2	12,0	2,4	24,0	8,9	0,0
Choco cookie bar	16	85,3	4,7	2,9	9,7	1,0	0,2
**Snack**							
Milk bread	40	138,0	2,1	1,3	20,4	3,8	0,9
Pork ham	31	61,7	1,6	0,6	0,2	11,8	0,0
Butter	12	89,2	9,8	6,7	0,1	0,1	0,0
**Dinner**							
Bread	21	56,5	0,8	0,1	10,1	1,9	0,6
Chicken	140	161,0	1,7	0,6	4,3	32,2	0,0
Milk cream	50	167,5	17,5	12,0	1,6	1,0	0,0
Noodles	40	141,6	0,6	0,1	28,8	4,4	1,6
Chocolate	5,5	30,8	1,87	1,1	3,19	0,264	0
**Total**		**2320**	**127**	**51**	**169**	**123**	**5**
**Breakfast**		671	42	11	50	28	1
**Lunch**		802	44	14	41	38	1
**Snack**		289	14	9	21	16	1
**Dinner**		557	22	14	48	40	2

Days 6 and 13 FMD

Day 6	gr	Kcal	Fat (g)	Saturated fat (g)	Carbohydrates (g)	Protein (g)	Fiber (g)
**Breakfast**							
Low-fat milk	200	92,0	3,2	2,2	9,6	6,2	0,0
Ham	60	61,2	1,9	0,7	4,3	6,8	0,0
Egg	68	102,7	7,5	0,0	0,3	8,5	0,0
Crackers	30	143,4	5,7	0,6	20,1	2,5	0,5
Sugar	14	56,0	0,0	0,0	14,0	0,0	0,0
**Lunch**							
Bread	21	56,5	0,8	0,1	10,1	1,9	0,6
3 cheese quiche	175	503,7	38,0	23,4	27,7	12,8	0,9
Bacon	53	188,5	17,1	6,9	0,5	8,0	0,0
Choco cookie bar	16	85,3	4,7	2,9	9,7	1,0	0,2
**Snack**							
Milk bread	40	138,0	2,1	1,3	20,4	3,8	0,9
Iberian chorizo	60	128,4	9,6	3,7	0,3	10,2	0,0
Butter	12	89,2	9,8	6,7	0,1	0,1	0,0
**Dinner**							
Bread	21	56,5	0,8	0,1	10,1	1,9	0,6
Beef	150	165,0	4,5	0,0	0,0	31,4	0,0
Potato omelette	200	316,0	17,8	2,8	28,0	8,8	2,9
Cheese	18	37,9	2,2	1,4	1,6	2,5	0,4
Chocolate	5,5	30,8	1,87	1,1	3,19	0,264	0
**Total**		**2251**	**128**	**54**	**160**	**107**	**7**
**Breakfast**		455	18	3	48	24	1
**Lunch**		834	61	33	48	24	2
**Snack**		356	22	12	21	14	1
**Dinner**		606	27	5	43	45	4

Days 7 and 14 FMD

Day 7	gr	Kcal	Fat (g)	Saturated fat (g)	Carbohydrates (g)	Protein (g)	Fiber (g)
**Breakfast**							
Low-fat milk	200	92,0	3,2	2,2	9,6	6,2	0,0
Salami	60	277,2	25,2	7,8	3,1	9,6	0,0
Egg	68	102,0	7,5	0,0	0,3	8,5	0,0
Crackers	30	143,4	5,7	0,6	20,1	2,5	0,5
Sugar	14	56,0	0,0	0,0	14,0	0,0	0,0
**Lunch**							
Bread	21	56,5	0,8	0,1	10,1	1,9	0,6
Pork chop	150	316,5	22,5	8,7	0,0	28,5	0,0
Russian salad	125	274,0	22,5	3,5	10,0	7,1	2,3
Snicker	18	86,6	4,1	1,4	11,0	1,5	0,0
Cheese	18	37,9	2,2	1,4	1,6	2,5	0,4
**Snack**							
Milk bread	40	138,0	2,1	1,3	20,4	3,8	0,9
Pork Ham	60	128,4	9,6	3,7	0,3	10,2	0,0
Butter	12	89,2	9,8	6,7	0,1	0,1	0,0
**Dinner**							
Bread	21	41,9	2,9	1,2	0,3	3,5	0,0
Chicken	140	161,0	1,7	0,6	4,3	32,2	0,0
Noodles	40	141,6	0,6	0,1	28,8	4,4	1,6
Butter	12	89,2	9,8	6,7	0,1	0,1	0,0
Chocolate	5,5	30,8	1,87	1,1	3,19	0,264	0
Milk cream	50	167,5	17,5	12	1,55	1	0

**Total**		**2430**	**150**	**59**	**139**	**124**	**6**
**Breakfast**		671	42	11	33	27	1
**Lunch**		734	50	14	31	39	3
**Snack**		356	22	12	21	14	1
**Dinner**		590	31	21	38	38	2

Days 1 and 8 WD

Day 1	gr	Kcal	Fat (g)	Saturated fat (g)	Carbohydrates (g)	Protein (g)	Fiber (g)
**Breakfast**							
Skim milk	200	80,0	0,6	1,0	10,6	7,8	0,0
Sugar	14	56,0	0,0	0,0	14,0	0,0	0,0
Rye bread	110	231,0	1,7	0,3	42,9	5,5	8,8
Strawberry jam	25	54,0	0,1	0,0	13,3	0,1	0,7
Honey	20	66,4	0,0	0,0	16,5	0,1	0,0
**Snack**							
Walnuts	30	178,5	19,0	0,0	1,0	4,2	1,6
Dates	40	149,2	0,1	0,0	35,1	0,4	3,2
**Lunch**							
Bread	35	89,3	0,8	0,2	17,9	3,3	1,4
Stewed beans	430	382,7	9,5	3,0	39,1	22,8	25,8
Mix salad	100	27,0	0,2	0,1	3,6	1,3	1,6
Oilve oil	5	45,0	5,0	0,0	0,0	0,0	0,0
Pear	205	118,9	0,2	0,0	28,7	0,6	1,0
**Snack**							
Chocolate soy drink	200	110,0	3,8	0,8	11,0	7,2	1,8
Cereal	40	149,6	0,3	0,0	32,0	3,7	1,9
Dehydrated apple	40	109,6	0,3	0,1	24,0	1,5	2,5
**Dinner**							
Bread	35	89,3	0,8	0,2	17,9	3,3	1,4
Brown rice	80	212,5	3,4	0,6	38,8	5,0	3,6
Chicken breast	70	80,5	0,8	0,3	2,2	16,1	0,0
Mix salad	100	27,0	0,2	0,1	3,6	1,3	1,6
Olive oil	5	45,0	5,0	0,0	0,0	0,0	0,0
Pineapple	113	63,3	0,0	0,0	14,7	0,6	1,1
**TOTAL**		**2365**	**52**	**7**	**367**	**85**	**58**
**Breakfast**		487	2	1	97	14	9
**Snack**		328	19	0	36	5	5
**Lunch**		663	16	3	89	28	30
**Snack**		369	4	1	67	12	6
**Dinner**		518	10	1	77	26	8

Days 2 and 9 WD

Day 2	gr	Kcal	Fat (g)	Saturated fat (g)	Carbohydrates (g)	Protein (g)	Fiber (g)
**Breakfast**							
Skim milk	200	80,0	0,6	1,0	10,6	7,8	0,0
Sugar	14	56,0	0,0	0,0	14,0	0,0	0,0
Rye bread	110	231,0	1,7	0,3	42,9	5,5	8,8
Peach jam	25	54,0	0,1	0,0	13,3	0,1	0,5
Honey	20	66,4	0,0	0,0	16,5	0,1	0,0
**Snack**							
Walnuts	30	178,5	19,0	0,0	1,0	4,2	1,6
Banana	150	133,5	0,5	0,0	30,0	1,8	5,1
**Lunch**							
Bread	35	89,3	0,8	0,2	17,9	3,3	1,4
Canned Tuna	60	47,4	0,4	0,1	0,3	10,8	0,0
Peas	140	172,2	1,5	0,1	23,8	10,6	11,2
Assorted vegetables	150	51,0	0,3	0,0	8,9	2,1	2,6
Tomato	150	28,5	0,2	0,0	5,3	1,4	1,7
Olive oil	5	45,0	5,0	0,0	0,0	0,0	0,0
Tangerines	180	72,0	0,0	0,0	16,2	1,4	3,4
**Snack**							
Vanilla soy drink	200	94,0	3,4	0,6	9,0	6,0	1,2
Cereal	40	152,4	0,6	0,1	31,6	4,8	1,2
Dehydrated mango	60	190,2	0,7	0,2	42,0	1,5	4,9
**Dinner**							
Bread	35	89,3	0,8	0,2	17,9	3,3	1,4
Beef	100	110,0	3,0	0,0	0,0	20,9	0,0
Quinoa	125	153,8	2,8	0,4	24,5	5,6	5,1
Assorted vegetables	150	51,0	0,3	0,0	8,9	2,1	2,6
Artichoke	60	24,6	0,3	0,0	3,6	1,1	2,4
Olive oil	5	45,0	5,0	0,0	0,0	0,0	0,0
Kiwi	200	104,0	1,0	0,0	21,2	2,2	3,8
**TOTAL**		**2319**	**48**	**3**	**359**	**97**	**59**
**Breakfast**		487	2	1	97	14	9
**Snack**		312	19	0	31	6	7
**Lunch**		505	8	1	72	30	20
**Snack**		437	5	1	83	12	7
**Dinner**		578	13	1	76	35	15

Days 3 and 10 WD

Day 3	gr	Kcal	Fat (g)	Saturated fat (g)	Carbohydrates (g)	Protein (g)	Fiber (g)
**Breakfast**							
Skim milk	200	80,0	0,6	1,0	10,6	7,8	0,0
Sugar	14	56,0	0,0	0,0	14,0	0,0	0,0
Rye bread	110	231,0	1,7	0,3	42,9	5,5	8,8
Turkey breast	30	23,4	0,2	0,1	0,2	5,3	0,0
Straw berry jam	20	66,4	0,0	0,0	16,5	0,1	0,0
**Snack**							
Walnuts	30	178,5	19,0	0,0	1,0	4,2	1,6
Dried apricots	40	109,6	0,3	0,1	24,0	1,5	2,5
**Lunch**							
Bread	35	89,3	0,8	0,2	17,9	3,3	1,4
Chikpeas with spinac	430	374,0	14,5	1,7	40,4	16,2	9,8
Assorted vegetables	150	64,5	0,4	0,1	12,3	1,8	2,6
Mixed salad	50	12,0	0,2	0,0	1,4	0,9	0,7
Olive oil	5	45,0	5,0	0,0	0,0	0,0	0,0
Pineapple	113	63,3	0,0	0,0	14,7	0,6	1,1
**Snack**							
Chocolate soy drink	200	110,0	3,8	0,8	11,0	7,2	1,8
Cereal	40	149,6	0,3	0,0	32,0	3,7	1,9
Dehydrated apple	20	73,0	0,1	0,0	16,6	0,2	2,4
**Dinner**							
Bread	35	89,3	0,8	0,2	17,9	3,3	1,4
Breast chicken	70	80,5	0,8	0,3	2,2	16,1	0,0
Corn	140	172,2	1,5	0,1	23,8	10,6	11,2
Assorted vegetables	150	64,5	0,4	0,1	12,3	1,8	2,6
Mixed salad	50	12,0	0,2	0,0	1,4	0,9	0,7
Olive oil	5	45,0	5,0	0,0	0,0	0,0	0,0
Tangerines	180	72,0	0,0	0,0	16,2	1,4	3,4
**TOTAL**		**2261**	**55**	**5**	**329**	**93**	**54**
**Breakfast**		457	2	1	84	19	9
**Snack**		288	19	0	25	6	4
**Lunch**		648	21	2	87	23	16
**Snack**		333	4	1	60	11	6
**Dinner**		535	9	1	74	34	19

Days 4 and 11 WD

Day 4	gr	Kcal	Fat (g)	Saturated fat (g)	Carbohydrates (g)	Protein (g)	Fiber (g)
**Breakfast**							
Skim milk	200	80,0	0,6	1,0	10,6	7,8	0,0
Sugar	14	56,0	0,0	0,0	14,0	0,0	0,0
Rye bread	110	231,0	1,7	0,3	42,9	5,5	8,8
Quince paste	40	90,0	0,0	0,0	22,3	0,1	0,0
**Snack**							
Walnuts	30	178,5	19,0	0,0	1,0	4,2	1,6
Dehydrated mango	60	190,2	0,7	0,2	42,0	1,5	4,9
**Lunch**							
Bread	35	86,1	5,7	0,6	3,7	4,3	1,3
Red lentil noodles	100	334	1,7	0,4	50	26	7,6
Mixed salad	50	15	0	0	3	1	1
Assorted vegetables	150	51,0	0,3	0,0	8,9	2,1	2,6
Artichoke	60	20,5	0,25	0	3	0,9	2
Olive oil	5	45,0	5,0	0,0	0,0	0,0	0,0
Pear	205	118,9	0,2	0,0	28,7	0,6	1,0
**Snack**							
Vanilla soy drink	200	94,0	3,4	0,6	9,0	6,0	1,2
Cereal	40	152,4	0,6	0,1	31,6	4,8	1,2
Dehydrated apple	20	73,0	0,1	0,0	16,6	0,2	2,4
**Dinner**							
Bread	35	89,3	0,8	0,2	17,9	3,3	1,4
Breast chicken	70	80,5	0,8	0,3	2,2	16,1	0,0
Quinoa	120	153,8	2,8	0,4	24,5	5,6	5,1
Assorted vegetables	150	51,0	0,3	0,0	8,9	2,1	2,6
Mixed salad	50	14,5	0,1	0,0	2,5	0,5	0,9
Olive oil	5	45,0	5,0	0,0	0,0	0,0	0,0
Plum	200	108,0	0,4	0,0	24,0	1,5	4,6
**TOTAL**		**2357**	**50**	**4**	**367**	**94**	**50**
**Breakfast**		457	2	1	90	13	9
**Snack**		369	20	0	43	6	6
**Lunch**		670	13	1	97	34	15
**Snack**		319	4	1	57	11	5
**Dinner**		542	10	1	80	29	15

Days 5 and 12 WD

Day 5	gr	Kcal	Fat (g)	Saturated fat (g)	Carbohydrates (g)	Protein (g)	Fiber (g)
**Breakfast**							
Skim milk	200	80,0	0,6	1,0	10,6	7,8	0,0
Sugar	14	56,0	0,0	0,0	14,0	0,0	0,0
Rye bread	110	231,0	1,7	0,3	42,9	5,5	8,8
Strawberry jam	25	54,0	0,1	0,0	13,3	0,1	0,5
Turkey breast	30	23,4	0,2	0,1	0,2	5,3	0,0
**Snack**							
Walnuts	30	178,5	19,0	0,0	1,0	4,2	1,6
Dates	40	149,2	0,1	0,0	35,1	0,4	3,2
**Lunch**							
Bread	35	89,3	0,8	0,2	17,9	3,3	1,4
Stewed lentils	420	283,8	11,6	1,7	28,8	12,5	7,3
Mixed salad	50	12,0	0,2	0,0	1,4	0,9	0,7
Cucumbrer	150	18,0	0,2	0,0	3,0	1,1	2,7
Olive oil	5	45,0	5,0	0,0	0,0	0,0	0,0
Banana	150	133,5	0,5	0,0	30,0	1,8	5,1
**Snack**							
Chocolate soy drink	200	110,0	3,8	0,8	11,0	7,2	1,8
Cereal	40	149,6	0,3	0,0	32,0	3,7	1,9
Dehydrated mango	60	190,2	0,7	0,2	42,0	1,5	4,9
**Dinner**							
Bread	35	89,3	0,8	0,2	17,9	3,3	1,4
Canned tuna	80	63,2	0,6	0,2	0,4	14,4	0,0
Brown rice	80	212,5	3,4	0,6	38,8	5,0	3,6
Tomato	150	28,5	0,2	0,0	5,3	1,4	1,7
Mixed salad	50	12,0	0,2	0,0	1,4	0,9	0,7
Olive oil	5	45,0	5,0	0,0	0,0	0,0	0,0
Tangerine	180	72,0	0,0	0,0	16,2	1,4	3,4
**TOTAL**		**2326**	**55**	**5**	**363**	**82**	**51**
**Breakfast**		444	2	1	81	19	9
**Snack**		328	19	0	36	5	5
**Lunch**		582	18	2	81	20	17
**Snack**		450	5	1	85	12	9
**Dinner**		522	10	1	80	26	11

Days 6 and 13 WD

**Day 6**	**gr**	**Kcal**	**Fat (g)**	**Saturated fat (g)**	**Carbohydrates (g)**	**Protein (g)**	**Fiber (g)**
Breakfast							
Skim milk	200	80,0	0,6	1,0	10,6	7,8	0,0
Sugar	14	56,0	0,0	0,0	14,0	0,0	0,0
Rye bread	110	231,0	1,7	0,3	42,9	5,5	8,8
Quince paste	40	90,0	0,0	0,0	22,3	0,1	0,0
**Snack**							
Walnuts	30	178,5	19,0	0,0	1,0	4,2	1,6
Dehydrated mango	60	190,2	0,7	0,2	42,0	1,5	4,9
**Lunch**							
Bread	35	89,25	0,84	0,21	17,85	3,325	1,4
Beef steak	100	110,0	3,0	0,0	0,0	20,9	0,0
Peas	140	172,2	1,5	0,1	23,8	10,6	11,2
Assorted vegetables	150	46,5	0,0	0,1	10,4	1,7	2,4
Mix salad	50	12,0	0,2	0,0	1,4	0,9	0,7
Olive oil	5	45	5	0	0	0	0
Kiwi	200	104,0	1,0	0,0	21,2	2,2	3,8
**Snack**							
Chocolate soy pudding	200	110	2,1	0,6	18,2	3,5	2,2
Cereal	40	152,4	0,6	0,1	31,6	4,8	1,2
Banana	150	133,5	0,5	0,0	30,0	1,8	5,1
**Dinner**							
Bread	35	89,25	0,84	0,21	17,85	3,325	1,4
Chicken breast	70	80,5	0,84	0,28	2,17	16,1	0
Corn	140	165,2	1,8	0,0	30,8	4,2	4,6
Assorted vegetables	150	46,5	0,0	0,1	10,4	1,7	2,4
Mixed salad	50	12	0,15	0,015	1,4	0,9	0,7
Olive oil	5	45	5	0	0	0	0
Plum	200	108	0,4	0	24	1,5	4,6
**TOTAL**		**2347**	**46**	**3**	**374**	**96**	**57**
**Breakfast**		457	2	1	90	13	9
**Snack**		369	20	0	43	6	6
**Lunch**		579	12	0	75	40	20
**Snack**		396	3	1	80	10	9
**Dinner**		457	8	0	69	24	12

Days 7 and 14 WD

Day 7	gr	Kcal	Fat (g)	Saturated fat (g)	Carbohydrates (g)	Protein (g)	Fiber (g)
**Breakfast**							
Skim milk	200	80,0	0,6	1,0	10,6	7,8	0,0
Sugar	14	56,0	0,0	0,0	14,0	0,0	0,0
Rye bread	110	231,0	1,7	0,3	42,9	5,5	8,8
Peach jam	25	54,0	0,1	0,0	13,3	0,1	0,5
Honey	20	66,4	0,0	0,0	16,5	0,1	0,0
**Snack**							
Walnuts	30	178,5	19,0	0,0	1,0	4,2	1,6
Dried apricots	40	109,6	0,3	0,1	24,0	1,5	2,5
**Lunch**							
Bread	35	89,3	0,8	0,2	17,9	3,3	1,4
Red lentil noodles	100	334,0	1,7	0,4	50,0	26,0	7,6
Tomato	150	28,5	0,2	0,0	5,3	1,4	1,7
Olive oil	5	45,0	5,0	0,0	0,0	0,0	0,0
Plum	200	108,0	0,4	0,0	24,0	1,5	4,6
Artichoques	60	24,6	0,3	0,0	3,6	1,1	2,4
Mix salad	150	51,0	0,3	0,0	8,9	2,1	2,6
**Snack**							
Chocolate soy pudding	200	110,0	2,1	0,6	18,2	3,5	2,2
Cereal	40	152,4	0,6	0,1	31,6	4,8	1,2
Dehydrated mango	60	190,2	0,7	0,2	42,0	1,5	4,9
**Dinner**							
Bread	35	89,3	0,8	0,2	17,9	3,3	1,4
Tuna	80	63,2	0,6	0,2	0,4	14,4	0,0
Quinoa	120	153,8	2,8	0,4	24,5	5,6	5,1
Assorted vegetables	150	51,0	0,3	0,0	8,9	2,1	2,6
Olive oil	5	45,0	5,0	0,0	0,0	0,0	0,0
Tangerines	180	72,0	0,0	0,0	16,2	1,4	3,4
**TOTAL**		**2383**	**43**	**4**	**391**	**91**	**54**
**Breakfast**		487	2	1	97	14	9
**Snack**		288	19	0	25	6	4
**Lunch**		680	9	1	110	35	20
**Snack**		453	3	1	92	10	8
**Dinner**		474	9	1	68	27	12

1. COLONIC CONTENT MEASUREMENT

MR imaging examinations of the colon were performed in the pretreatment and treatment phases (both on the challenge diet) using a 1.5-T MR imaging system (Aera; Siemens Healthcare, Erlangen, Germany) with two six-channel phased-array abdominal coils to cover the whole abdomen. The abdomen was imaged obtaining a T2-weighted HASTE sequence in the coronal plane (1400 ms repetition time, 90 ms echo time, 3.5 mm slice thickness, 180º flip angle, and 256x256 matrix resolution) during two apneas of 20 s each and a T1-weighted VIBE Fat-Sat sequence in the coronal plane (3.71 ms repetition time, 1.66 ms echo time, 1.5 mm slice thickness, 10 flip angle and 320x189 matrix resolution) in one apnea of 12 s. No drugs or contrast were used.

Analysis of the images was made using an original software developed for this purpose[1]. The program allows semiautomatic segmentation of the colon on the images using a region-growing-based algorithm. First, an anisotropic contrast enhancement filter is applied to enhance the colon's boundary without loss of inside detail. Then, seed points are placed that expand depending on the gray-level mapping defined by the window-level setting of the images. A toolkit was developed to facilitate colonic segmentation, which permits enlarging or reducing the segmentation obtained by the region-growing algorithm. Colonic segmentation was correlated in T2 and T1 images. Nongaseous colonic content was measured in T1 colonic images, and gaseous colonic content was measured by subtracting T1 from T2 colonic volumes. A three-dimensional reconstruction program with 360º rotation over the three dimensions was used to facilitate the measurement of volumes in selected regions of the colon[2].

2. UNTARGETED METABOLOMIC ANALYSIS

Enzymatic treatment of urine samples

Urine samples (0.5 mL) were incubated with 25 μL β-glucuronidase and 3 mg sulfatase for 24 h at 37 ºC with gentle agitation. After incubation, samples were extracted twice with 2 mL ethyl acetate and evaporated, further re-dissolved in 2 mL MeOH, and analyzed by UPLC-ESI-QTOF-MS.

UPLC-ESI-QTOF-MS analysis

The analyses were carried out using an Agilent 1290 Infinity series LC system coupled to a 6550 I-Funnel Accurate-Mass QTOF (Agilent Technologies, Waldbronn, Germany) with a dual electrospray ionization interface (ESI-Jet Stream Technology) for simultaneous spraying of a mass reference solution that enabled continuous calibration of detected *m/z* ratios. Urine samples (with or without enzymatic treatment) were injected into a reverse-phase column, a Poroshell 120 EC column (3 x 100 mm, 2.7 μm) (Agilent Technologies), operating at 30 ºC and a flow rate of 0.5 mL/min. The mobile phases used were acidified water (0.1% formic acid) (Phase A) and acidified ACN (0.1% formic acid)(Phase B). Compounds were separated using the following gradient conditions: 0–3 min, 5–18% phase-B; 3–10 min, 18–50% phase-B; 10–13 min, 50–90% phase-B. Finally, phase B content was returned to the initial conditions (5%) for 1 min, and the column re-equilibrated for two more minutes. Data were acquired using the Mass Hunter Workstation software (version B.08.00, Service Pack 1, Agilent Technologies). The system was operated using both negative and positive ion polarity. Data were acquired in centroid and profile mode, with a data storage threshold of 5000 absorbance for MS and 5000 absorbance for MS/MS. The

operating conditions were as follows: gas temperature of 280 ºC, drying nitrogen gas of 9 L/min, nebulizer pressure of 45 psi, sheath gas temperature of 400 ºC, sheath gas flow of 12 L/min, a capillary voltage of 3500 V, nozzle voltage of 500 V, fragmentor voltage of 100 V, skimmer of 65 V and octopole radiofrequency voltage of 750 V. TOF spectra acquisition rate/time was 1.5 spectra/s and 666.7 ms/spectrum respectively, and transients/spectrum were 5484. The mass range was between 50 and 1100 *m/z*. At the beginning of the batch, the instrument was calibrated to assure mass accuracy during the MS analysis using a mixture of reference compounds (Tuning Mix). Continuous internal calibration was performed during analyses using signals *m/z* 112.9855 and *m/z* 1033.9881 in negative polarity and *m/z* 121.0509 and *m/z* 922.0098 in positive polarity. Auto recalibration reference-mass parameters were a detection window of 100 ppm and a minimum height of 1000 counts. MS/MS conditions were collision energy 20 eV and acquisition time 100 ms/spectrum. Data were processed using the Mass Hunter Qualitative Analysis software (version B.08.00, Service Pack 1, Agilent Technologies).

As there was no specific target analytes (untargeted analysis), generic settings were applied to obtain as many compounds as possible. QTOF-MS offers high selectivity, sensitivity, resolution, and high mass accuracy, providing a powerful tool for complex samples' metabolic profiling. All samples were injected in the same batch, and the order of sample injection was randomized to avoid sample bias. A mixture with one replicate of each samples group was used as 'quality control' (QC) and was injected at the beginning and the end of the batch. Besides, MeOH injections were included every three samples as a blank run to avoid the carry-over effect.

Data pretreatment

Batch Feature Finding: MassHunter Profinder

Feature extraction was carried out on Agilent Profinder B.06.00 (Agilent Technologies, Waldbronn, Germany), a stand-alone feature extraction program for LC/MS-based profiling analyses. Profinder was optimized to extract features from large data sets and provide an intuitive user interface to inspect and review each feature across the files associated with the data set. Extracted ion chromatograms and mass spectral data related to each feature were revised and compared simultaneously, and scored by the software.

Once the data files were selected, the recursive feature extraction workflow algorithm was selected, and then, the method for the feature extraction algorithm was edited and reviewed. To find compounds by Molecular Features (MFs) was carried out using a pre-filter to take peaks with a height greater or equal to 10000 counts, allowing only –H and +HCOO as negative ions species and +H as positive ions, a peak spacing tolerance of 0.0025 *m/z* plus 7.0 ppm, and charge states limited to a maximum of 2. The file obtained with this pre-processing was created for each sample as a CEF file. The final files were exported into the Mass Profiler Professional (MPP) software package (revision B.14.09.01, Agilent Technologies, Santa Clara, CA, USA) for statistical analysis.

A new experiment typed as unidentified was created in MPP, and significance testing and fold change were done. The data source used for the experiment was MassHunter Qual, and the organism was *Homo Sapiens*. In the second step, data were imported from files. The following steps were sample reordering and experiment grouping. After data were imported, several filtering options were

applied, such as a minimum absolute abundance of 5000 counts, minimum mass filtering of 70 and maximum mass filtering of 1200, numbers of ions required greater or equal to 2, and the option to all charge states forbidden.

Alignment

The next step was the alignment of parameters, where unidentified compounds were aligned across the different samples based on their retention times' tolerance and the mass spectral similarity. Compounds from different samples with the same *m/z* (mass tolerance window: 5 ppm, 2mDa) and retention time (tolerance window: 0.15 min) were considered the same.

Normalization and Baseline Transformation

Next, the baselining pre-processing options were selected. The goal of normalization is to limit systematic non-biological variation to reveal real biological variation. Normalization steps were performed 'within samples' whereas baselining was 'per entity'. Using baseline transformation improved both visualization of abundance pattern similarities and grouping of masses with similar abundance patterns in clustering analysis. Data were transformed to the log 2 scale and centered to the median across samples set to lower the relatively large differences in the respective MFs abundance. All compounds were treated equally regardless of their abundance.

Quality Control on Samples and Entities

Quality control can be performed at both the sample and mass levels to eliminate low-quality samples and unreliable measurements. Carrying over low-

quality samples and masses could limit statistically significant findings or informative clustering results.

'Filter on frequency, on flags, and by abundance' criteria were applied to reduce the dimensionality of the data and select the most representative compounds, which are rarely detected (therefore not very reliable), and filter out entities with non-reproducible measurements within a condition. Only the MFs present in at least 80% of replicates in at least one condition were considered. This entity filtering allowed creating a higher quality data set so that the following multivariate analysis should be more significant.

Multivariate and statistical analysis in the metabolomic analysis

In a second step, statistical comparisons were performed between the Western-type diet (WD) and the fiber-enriched Mediterranean-type diet (FMD). Student's t-test unpaired analyses were applied with a level of significance of $p < 0.05$ with Bonferonni Holm Family-wise Error Rate (FWER) multiple testing corrections and a fold-change cut-off of 2.0. Student's t-test was mainly focused on searching for the null hypothesis's statistical significance between WD and FMD. A list of compounds that significantly differed between groups was generated. Both supervised methods (PLS-DA), suitable for classification, and unsupervised methods (PCA, Clustering) appropriate for pattern identification, were used to visualize statistical results, using the final list of statistically significant MFs.

Compound identification: Molecular structure correlator

The MassHunter MSC (Molecular Structure Correlator) program was used. This software accurately correlated mass MS/MS fragment ions for a compound of interest with one or more proposed molecular structures. Then, each selected formula was used to retrieve one or multiple possible structures from several on-line database and libraries, including the MassHunter compound database (PCDL), METLIN/ChemSpider/KEGG/Lipid Maps, etc., and scored how well each candidate structure correlated with the MS/MS spectrum.

The validation of tentatively identified compounds by the MassHunter program was carried out (whenever possible) by chromatographic comparison with authentic standards. When authentic standards were not available, indirect validation was tried according to the fragmentation by MS/MS and metabolites' elution order.

References

1. Bendezu RA, Mego M, Monclus E, et al. Colonic content: effect of diet, meals, and defecation. Neurogastroenterol Motil 2017;29:e12930.

2. Bendezu RA, Barba E, Burri E, et al. Colonic content in health and its relation to functional gut symptoms. Neurogastroenterol Motil 2016;28:849-854.

Table S1. Metabolites significantly different in urine after consumption of FMD and WD.

RT (min)	Ionisation mode	Exact Mass	Probable elemental formula	Initial tentative identification	Score (%)	Availability of standard	Metabolite confirmed	Increase (-fold)
				Increased metabolites after FMD				
0.9	+	76.0757	$CH_3 N_3 O$	Trimethylamine *N*-oxide (TMAO)	100.0	YES	TMAO	1.5
3.3	+	381.0800	$C_{15} H_{18} O_{10}$	3,4-Dihydroxyphenyl propionic acid 3-*O*-glucuronide	96.9	NO	NC*	2.7
4.2	+	281.1134	$C_{13} H_{16} N_2 O_5$	L-Aspartyl phenylalanine	100.0	YES	L-aspartyl phenylalanine	2.4
4.8	+	134.0603	$C_8 H_7 N O$	Hydroxyindole derivative	97.6	YES	5-hydroxyindole	2.0
5.4	-	250.0718	$C_{12} H_{13} N O_5$	*N*-Feruloylglycine	98.1	NO	NC	2.0
10.0	-	567.3167	$C_{30} H_{48} O_{10}$	• Deoxycholate 3-glucuronide<br>• Hyodeoxycholate 3-glucuronide<br>• Chenodeoxycholate 3-glucuronide<br>• Ursodeoxycholate 3-glucuronide	96.5	NO	Deoxycholic acid*	2.1
				Increased metabolites after WD				
2.3	+	218.1395	$C_{10} H_{20} N O_4$	Propionyl-L-carnitine	100.0	YES	Propionyl-L-carnitine	2.2
2.3	-	216.0332	$C_8 H_{11} N O_4$	*p*-Tyramine 3-*O*-sulfate	97.6	NO	*p*-Tyramine*	2.4
6.8	+	242.2122	$C_{14} H_{27} N O_2$	Sphingosine	95.3	YES	NC	17.5
8.4	-	541.2644	$C_{27} H_{42} O_{11}$	Cortolone 3-glucuronide	96.5	NO	Cortolone*	2.0
9.7	-	467.2641	$C_{25} H_{40} O_8$	• 18-acetoxy-PGF2α-11-acetate methyl ester<br>• 3-α-Androstanediol glucuronide	95.9	NO	PGF2α*	2.0

*Confirmation (or not) of the unconjugated metabolite (commercially available) after treatment of the urine with glucuronidase/sulfatase; FMD, fibre-enriched Mediterranean-type diet (FMD); RT, retention time; WD, Western-like diet; NC, not confirmed.

Table S2. Characteristic microbial gene families present in individuals showing a robust and non-robust microbiota (see **Figure 2**).

Statistically significant (*p < 0.05*) microbial gene families present in individuals showing a robust and non-robust microbiota
8319 significant gene families associated to specific microbial species

Robust microbiota: 7628 gene families

Most abundant species (number of genes):

- ❑ *Bacteroides uniformis* (2347)
- ❑ *Bacteroides uniformis* CAG 3 (1712)
- ❑ *Bacteroides vulgatus* (1597)
- ❑ *Bacteroides vulgatus CAG 6* (952)
- ❑ *Parabacteroides distasonis* (176)
- ❑ *Bacteroides caccae* (147)
- ❑ *Parabacteroides merdae* (119)
- ❑ *Bacteroides ovatus* (90)
- ❑ *Ruminococcus bromii* (73)
- ❑ *Bacteroides xylanisolvens* (52)

Non-robust microbiota: 691 gene families

Most abundant species (number of genes):

- ❑ *Bacteroides uniformis* CAG 3 (237)
- ❑ *Bacteroides uniformis* (173)
- ❑ *Ruminococcus bromii* (71)
- ❑ *Bacteroides vulgatus* (58)
- ❑ *Gemmiger formicilis* (36)
- ❑ *Bacteroides vulgatus CAG 6* (31)
- ❑ *Bacteroides ovatus* (21)
- ❑ *Bacteroides caccae* (15)
- ❑ *Bacteroides thetaiotaomicron* (13)
- ❑ *Parabacteroides distasonis* (13)

Robust microbiota: 1322 genes with known functions associated

Most represented functions (number of genes):

- ❑ Conserved domain protein (9)
- ❑ Peptidyl-prolyl cis-trans isomerase (8)
- ❑ TonB-dependent receptor plug domain protein (8)
- ❑ Two component, sigma54 regulator, Fis family (8)
- ❑ ABC transporter ATP-binding protein (7)
- ❑ Glycoside hydrolase family 43 (7)
- ❑ Transcriptional regulator AraC family (7)
- ❑ Transposase (7)
- ❑ Tyrosine type site-specific recombinase (7)
- ❑ ABC-2 type transporter (6)

Non-robust microbiota: 134 genes with known functions associated

Most represented functions (number of genes):

- ❑ ABC transporter ATP-binding protein (2)
- ❑ Beta-lactamase (2)
- ❑ Kinase PfkB family (2)
- ❑ L-aspartate oxidase (2)
- ❑ Oligopeptide transporter OPT family (2)
- ❑ Protein GrpE (2)
- ❑ Sodium/alanine symporter (2)

Table S3. Distinctive microbial metabolic pathways expressed in individuals showing a robust and non-robust microbiota (see **Figure 2**).

Statistically significant (*$p < 0.05$*) microbial metabolic pathways expressed in individuals showing a robust and non-robust microbiota

250 significant gene families associated with specific microbial species

Robust microbiota: 164 metabolic pathways

Most abundant species (number of pathways):

- ❑ *Bacteroides vulgatus* (51)
- ❑ *Bacteroides vulgatus* CAG 6 (39)
- ❑ *Bacteroides uniformis* CAG 3 (34)
- ❑ *Bacteroides uniformis* (30)
- ❑ *Flavonifractor plautii* (2)
- ❑ *Prevotella copri* (2)

Most represented pathways (number of pathways):

- ❑ Glycolysis IV (6)
- ❑ Adenosine ribonucleotides de novo biosynthesis (5)
- ❑ Calvin-Benson-Bassham cycle (5)
- ❑ Pyruvate fermentation to isobutanol (5)
- ❑ 5-aminoimidazole ribonucleotide biosynthesis I (4)
- ❑ 5-aminoimidazole ribonucleotide biosynthesis II (4)
- ❑ Coenzyme A biosynthesis II (4)
- ❑ Folate transformations II (4)
- ❑ GDP-mannose biosynthesis (4)
- ❑ Glycolysis I (from glucose 6-phosphate) (4)

Non-robust microbiota: 86 metabolic pathways

Most abundant species (number of genes):

- ❑ *Bacteroides caccae* (36)
- ❑ *Gemmiger formicilis* (15)
- ❑ *Bacteroides vulgatus* CAG 6 (8)
- ❑ *Parabacteroides distasonis* (6)
- ❑ *Bacteroides thetaiotaomicron* (5)
- ❑ *Bacteroides ovatus* (4)
- ❑ *Bacteroides uniformis* (3)
- ❑ *Bacteroides vulgatus* (2)
- ❑ *Blautia obeum* (2)
- ❑ *Blautia wexlerae* (2)

Most represented pathways (number of pathways):

- ❑ Glycolysis IV (4)
- ❑ Guanosine ribonucleotides de novo biosynthesis (4)
- ❑ dTDP-L-rhamnose biosynthesis I (3)
- ❑ Phosphopantothenate biosynthesis I (3)
- ❑ 5-aminoimidazole ribonucleotide biosynthesis II (2)
- ❑ Adenine and adenosine salvage III (2)
- ❑ Adenosine deoxyribonucleotides de novo biosynthesis II (2)
- ❑ Adenosine ribonucleotides de novo biosynthesis (2)
- ❑ Coenzyme A biosynthesis II (2)
- ❑ Folate transformations II (2)

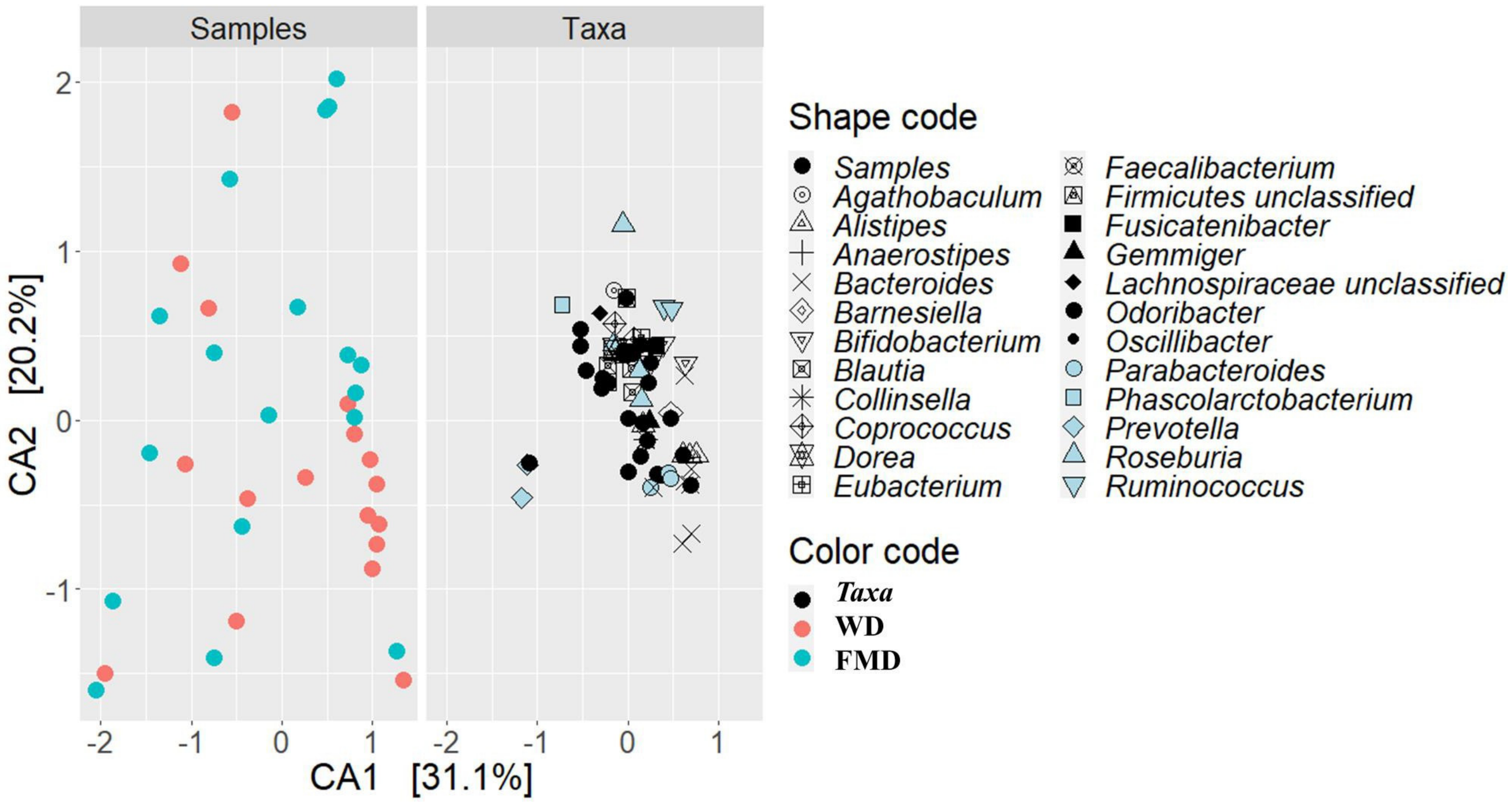

Figure S1. Canonical correspondence analysis (**CCA**) at genus level of samples obtained after Western-type Diet (**WD**) or Fiber-enriched Mediterranean-type Diet (**FMD**) interventions. **CA**: correspondence analysis. Some samples were grouped similar to principal coordinate analysis (**PCoA**) (See **Supplementary material Figure S4**). Differences in sample distribution might be due to additional factors other than diet, highlighting the importance of inter-individual variability. The percentage of variance explained by each CA is indicated in the axis.

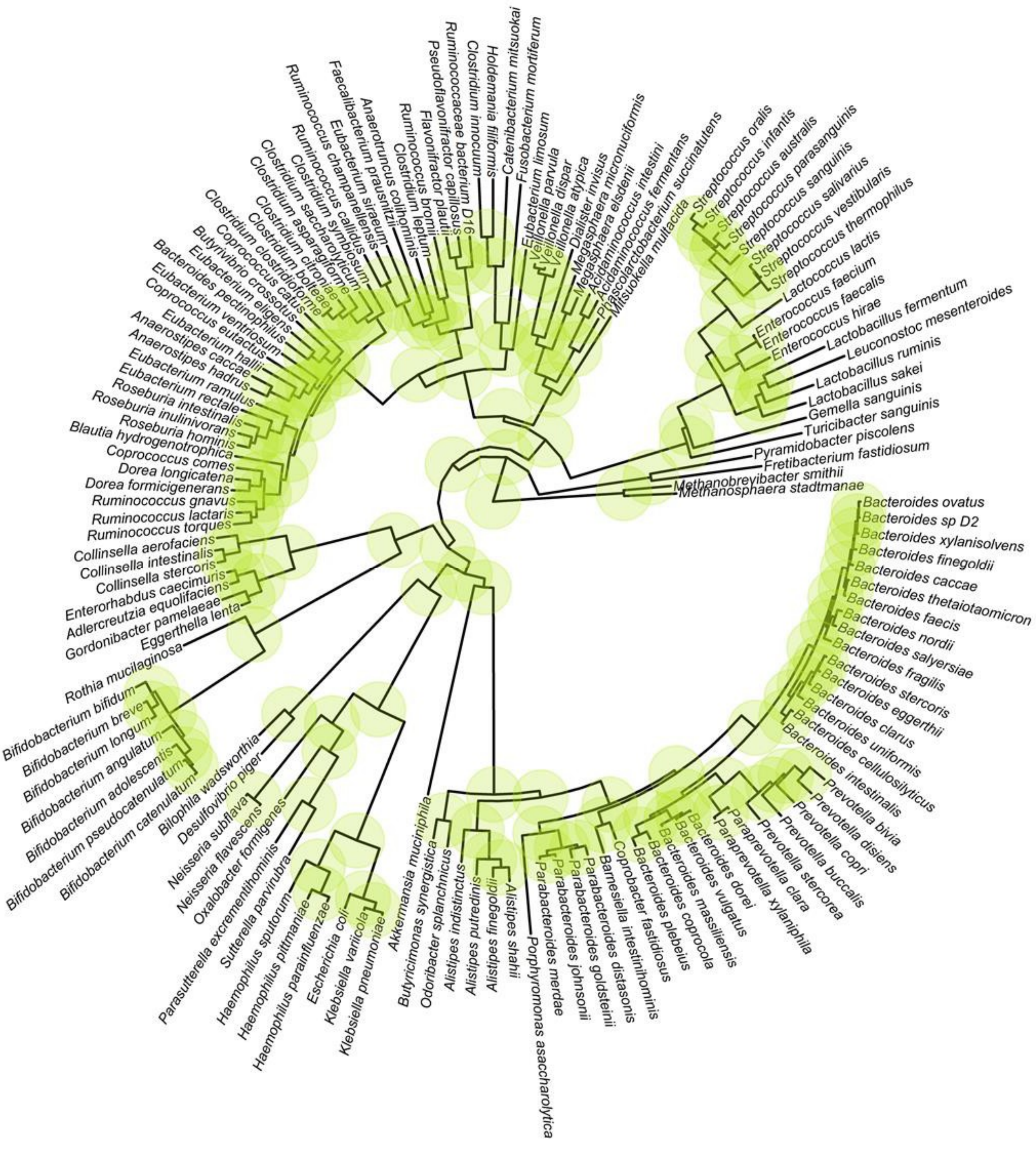

Figure S2. Cladogram calculated from phylogenetic distances of microbial species found in metagenomic samples from participants. Among species analysed, *Anaerostipes hadrus* showed high abundances after Fiber-enriched Mediterranean-type Diet (**FMD**) (for further details, see **Figure 1**).

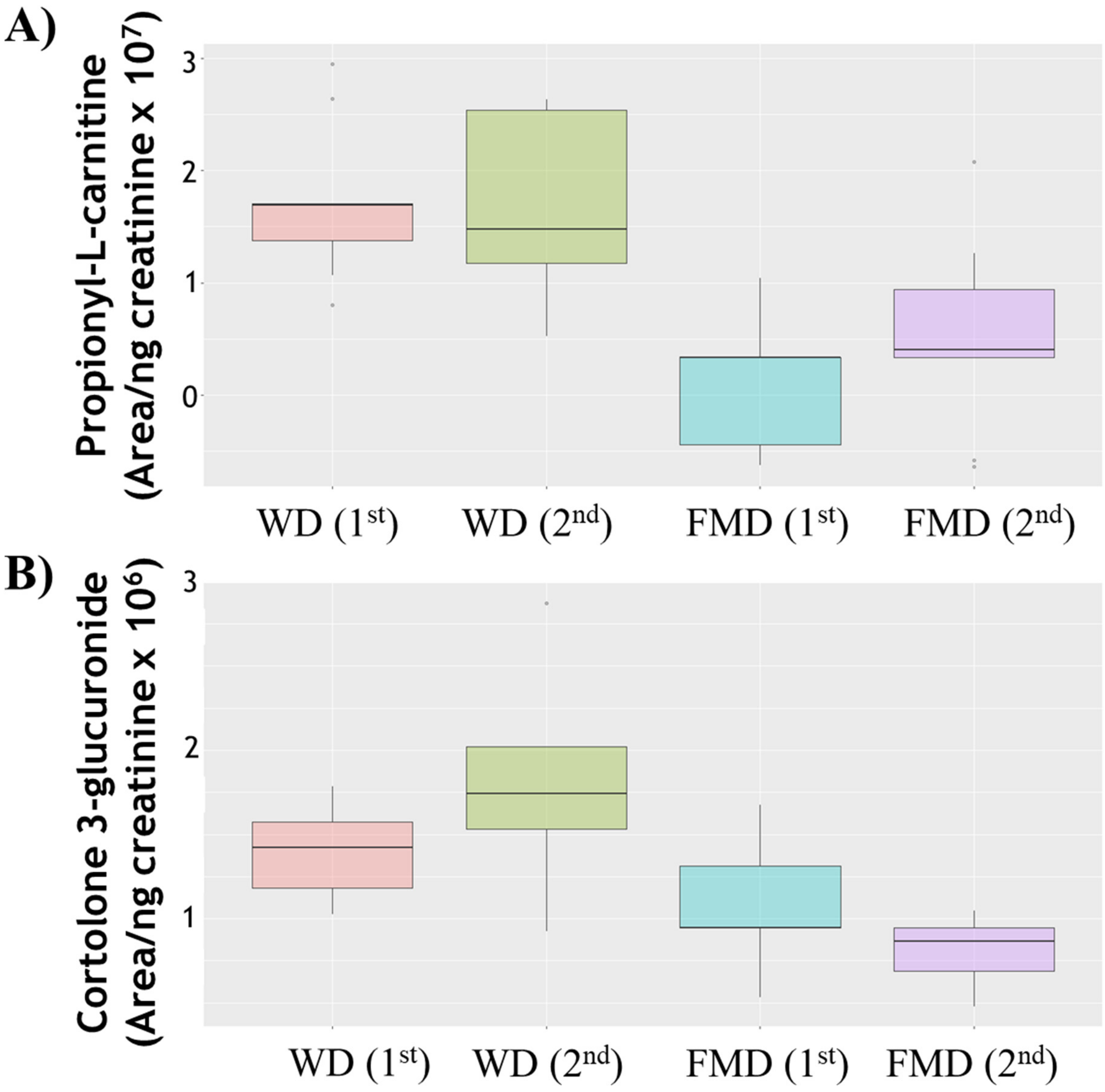

Figure S3. Carry-over effects observed for propionyl-L-carnitine (**A**) and Cortolone 3- glucuronide (**B**) levels in samples obtained after Western-type Diet (**WD**) or Fiber- enriched Mediterranean-type Diet (**FMD**) intervention at 1st or 2nd period.

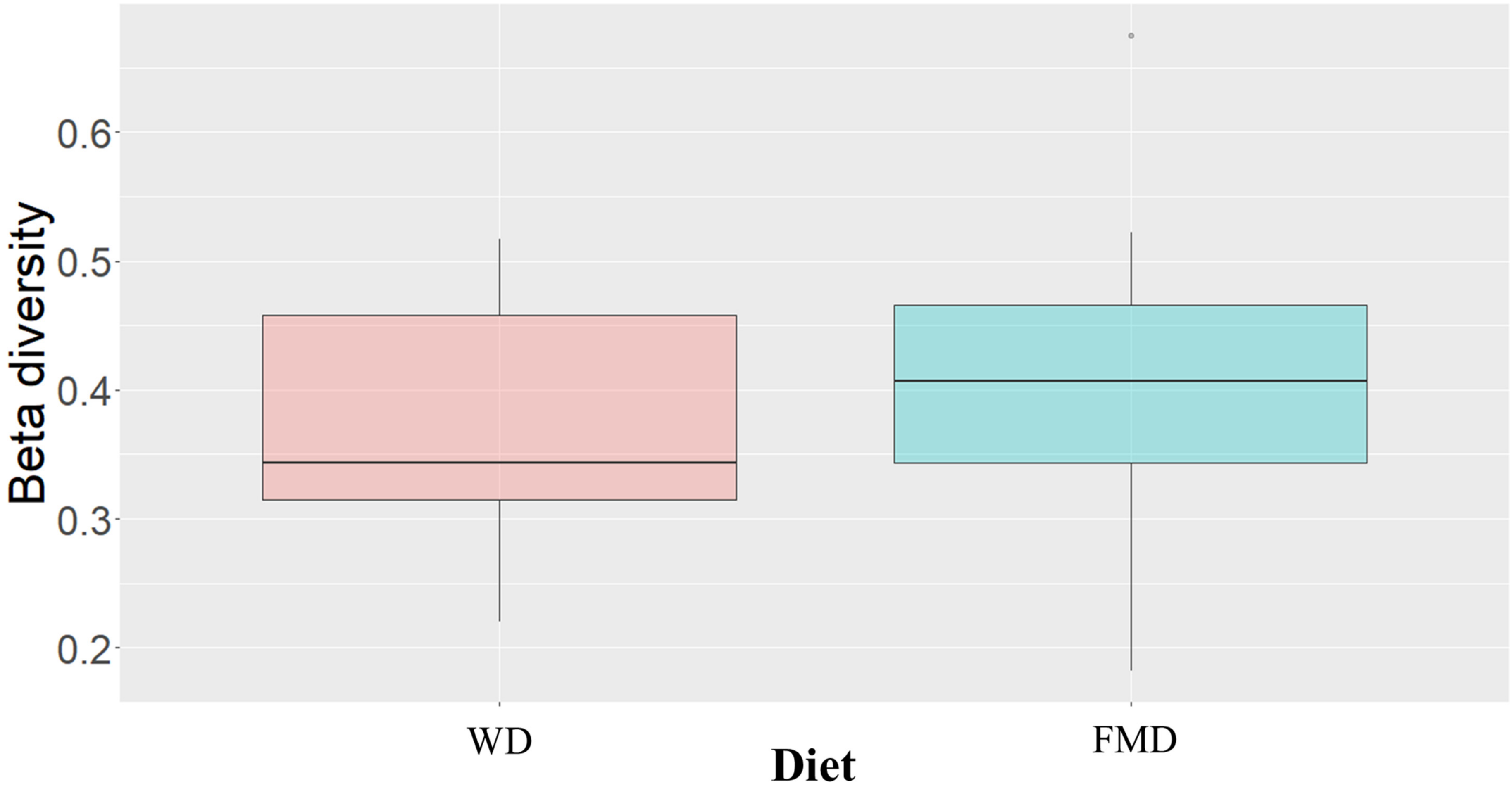

Figure S4. Beta-diversity analysis of metabolic pathways determined in samples obtained after Western-type Diet (**WD**) or Fiber-enriched Mediterranean-type Diet (**FMD**) interventions. The Bray-Curtis method was selected for the calculation. A total of 27 metabolic pathways showed significantly higher expresión after FMD intervention.

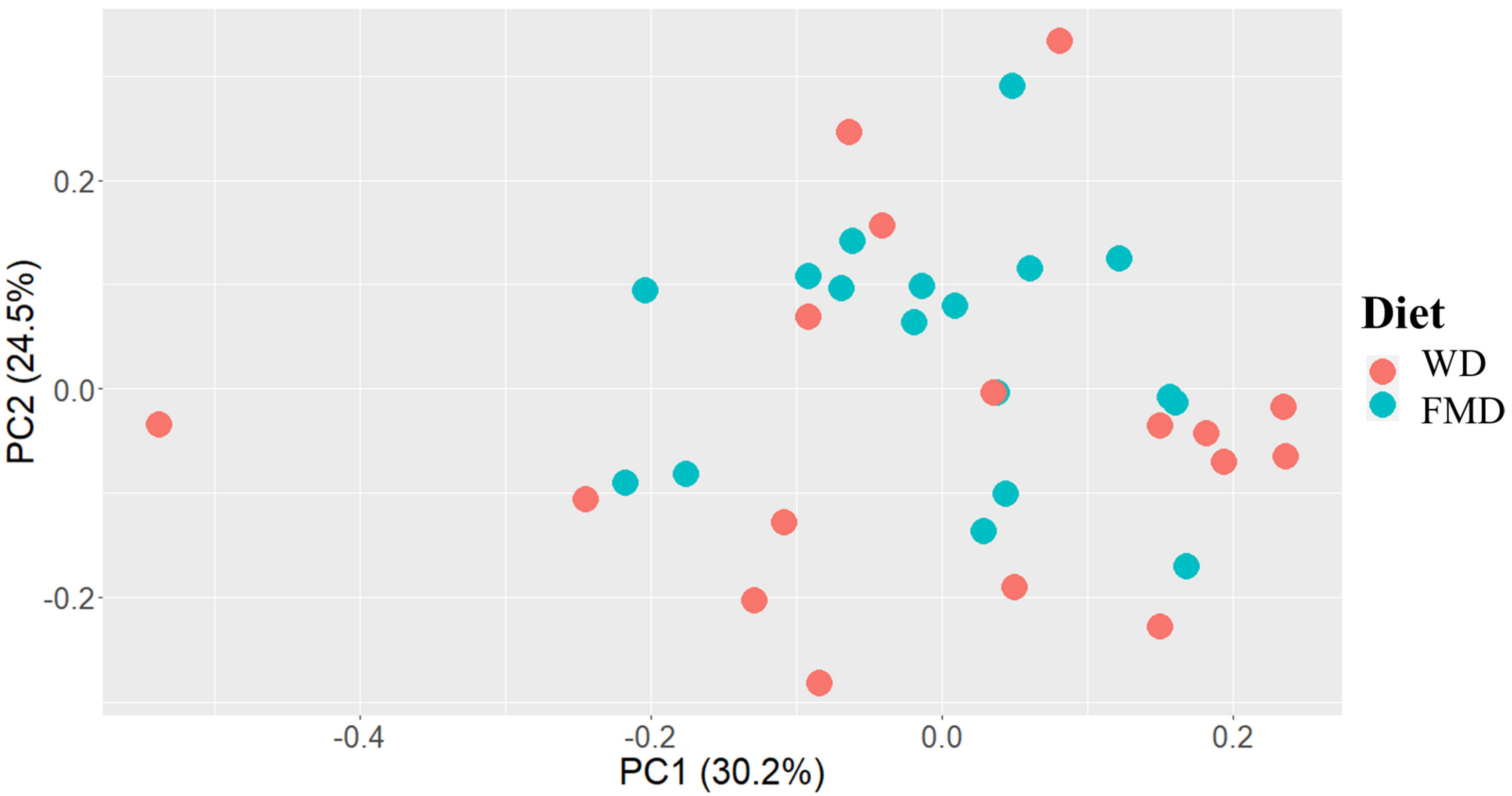

Figure S5. Principal coordinates analysis (**PCoA**) of taxa present in samples obtained after Western-type Diet (**WD**) or Fiber-enriched Mediterranean-type Diet (**FMD**) interventions. No significant differences can be observed in the global taxonomic profiles of participants according to the diet. **PC:** principal coordinate. The percentage of variance explained by each PC is indicated in the axis.

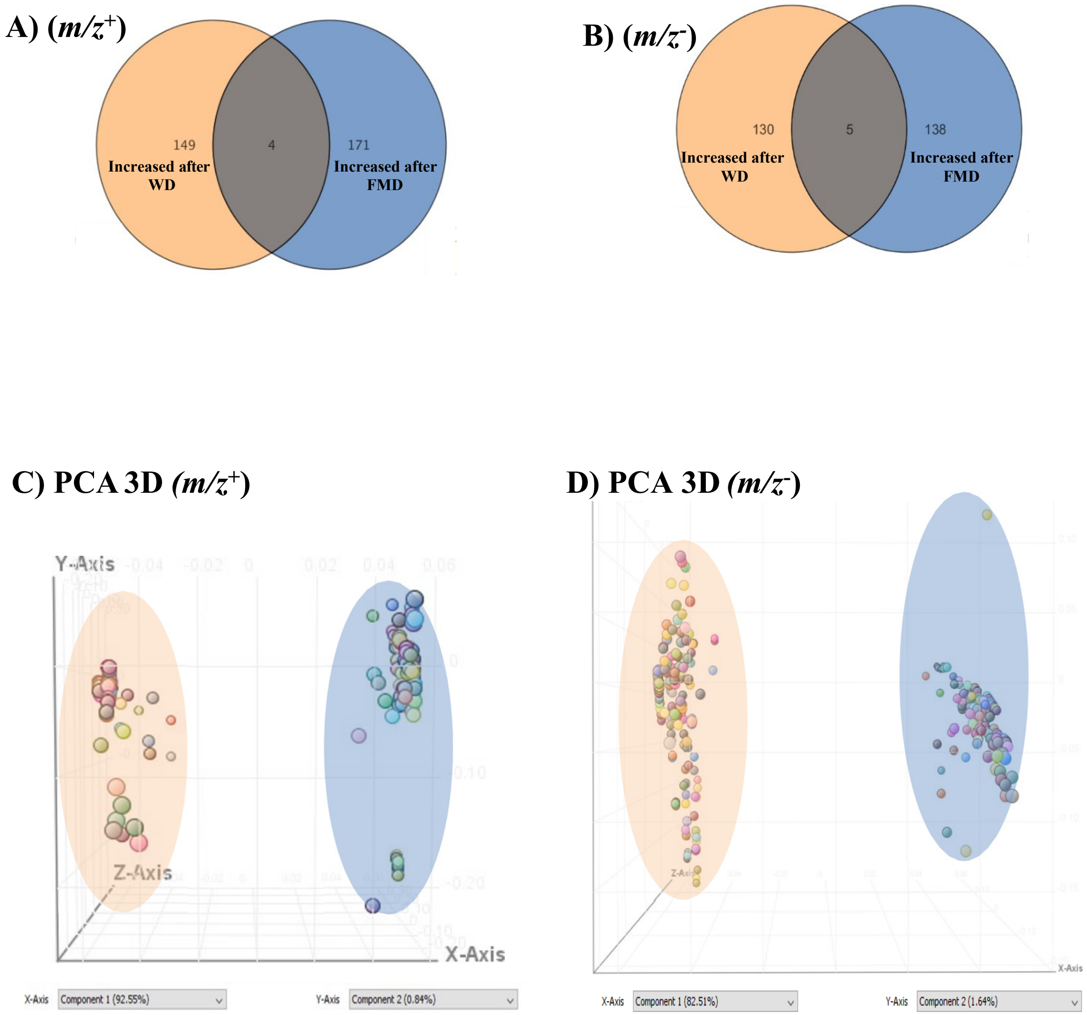

Figure S6. Venn diagrams (**A, B**) and three-dimension Principal Component Analyses (PCA) (**C, D**) showing those metabolites significantly increased (≥2-fold) after WD (orange colour) and after FMD (blue colour) in UPLC-QTOF-MS positive (**A, C**) and negative (**B, D**) detection modes.

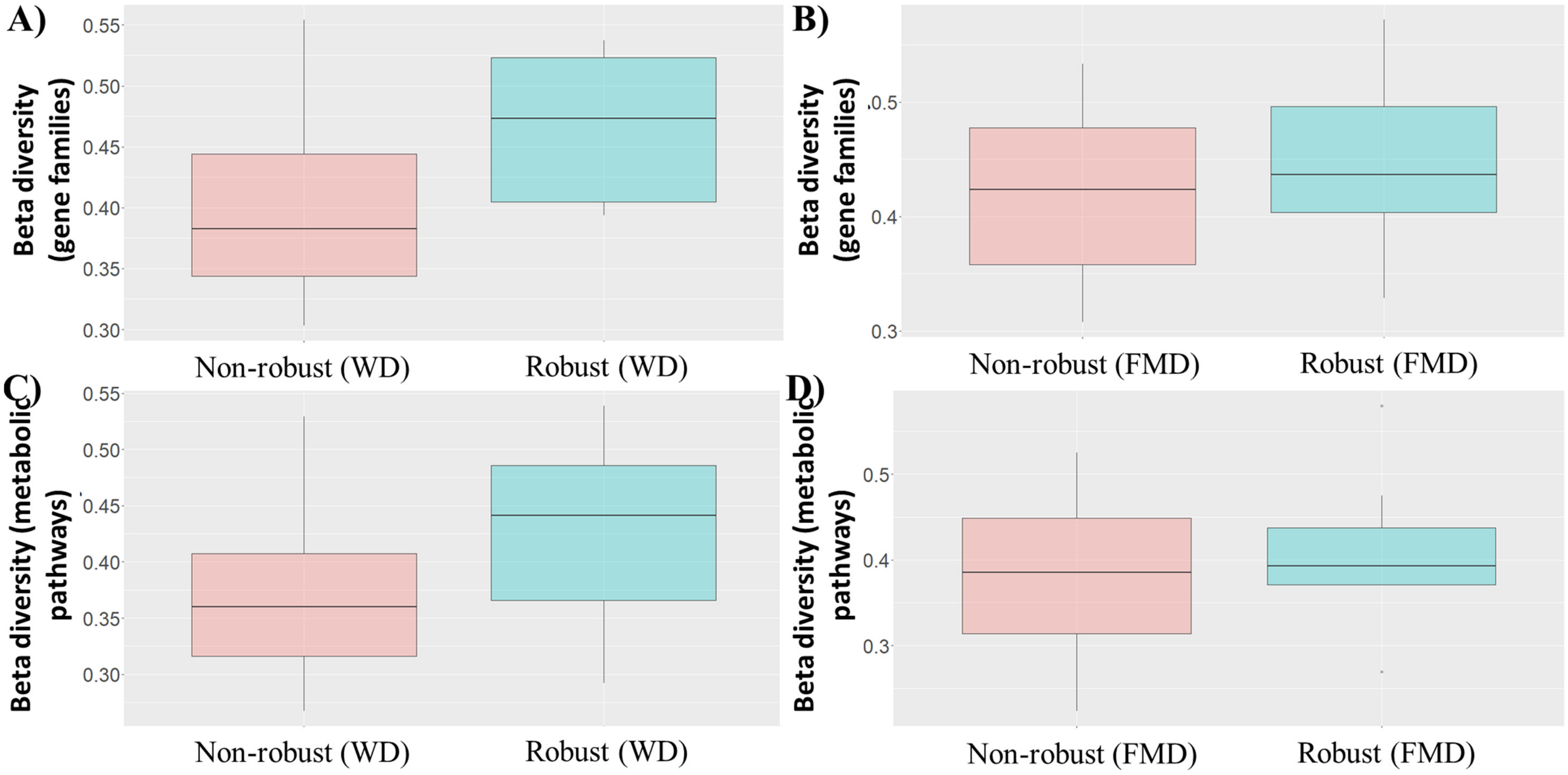

Figure S7. Beta-diversity analysis of gene families (**A, B**) and metabolic pathways (**C, D**) determined in samples from participants showing a robust and non-robust microbiota after Western-type Diet (**WD**) or Fibre-enriched Mediterranean-type Diet (**FMD**) interventions (for further details, see **Figure 2**). The Bray-Curtis method was selected for the calculation. Beta-diversity values were slightly higher in robust microbiotas that did not suffer any relevant change after **WD (A, C)**.

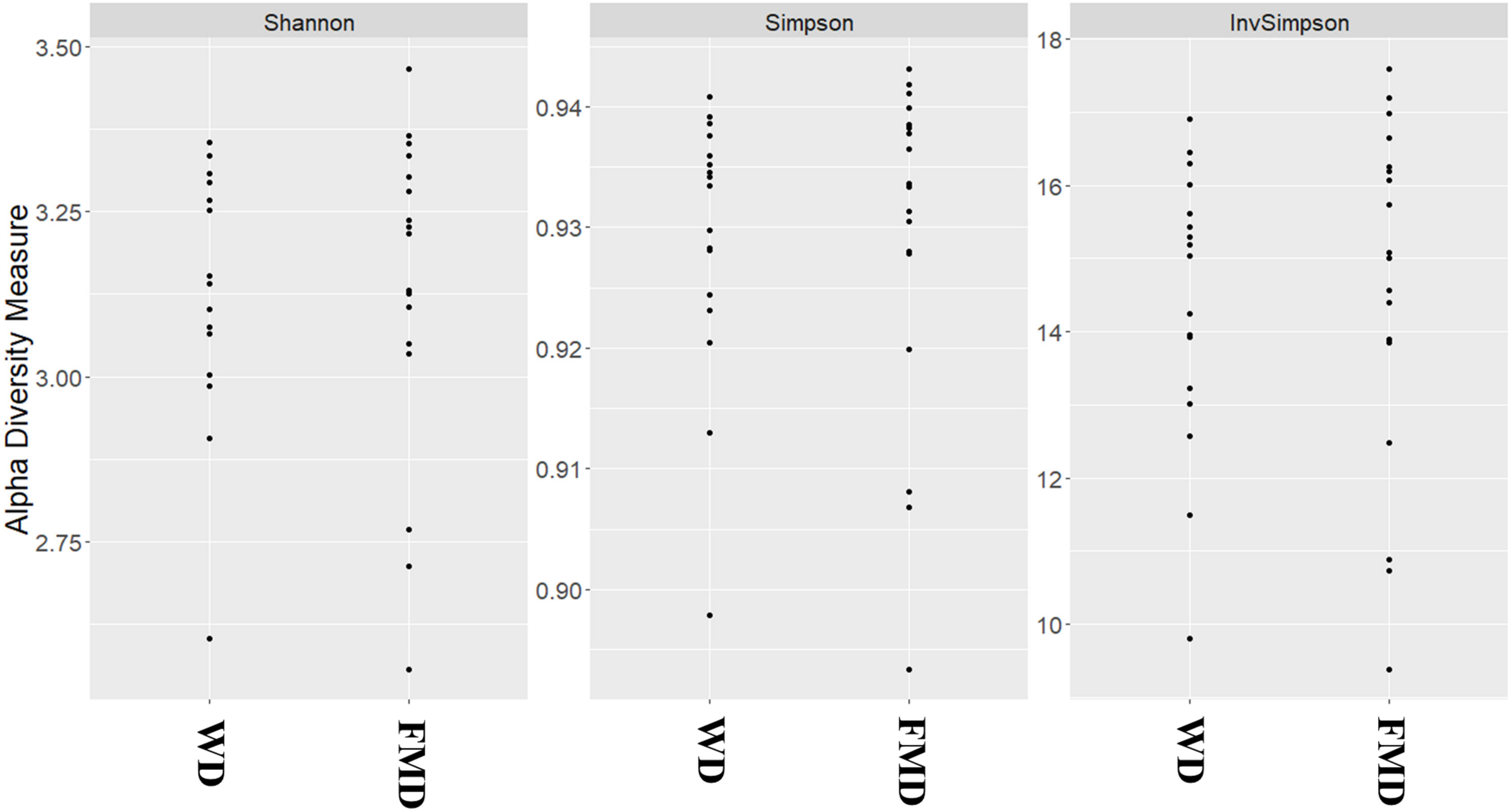

Figure S8. Comparison of different alpha-diversity indicators (Shannon, Simpson and Inverse Simpson) of the relative abundance of taxa determined after Western-type Diet (**WD**) or Fiber-enriched Mediterranean-type Diet (**FMD**) interventions.

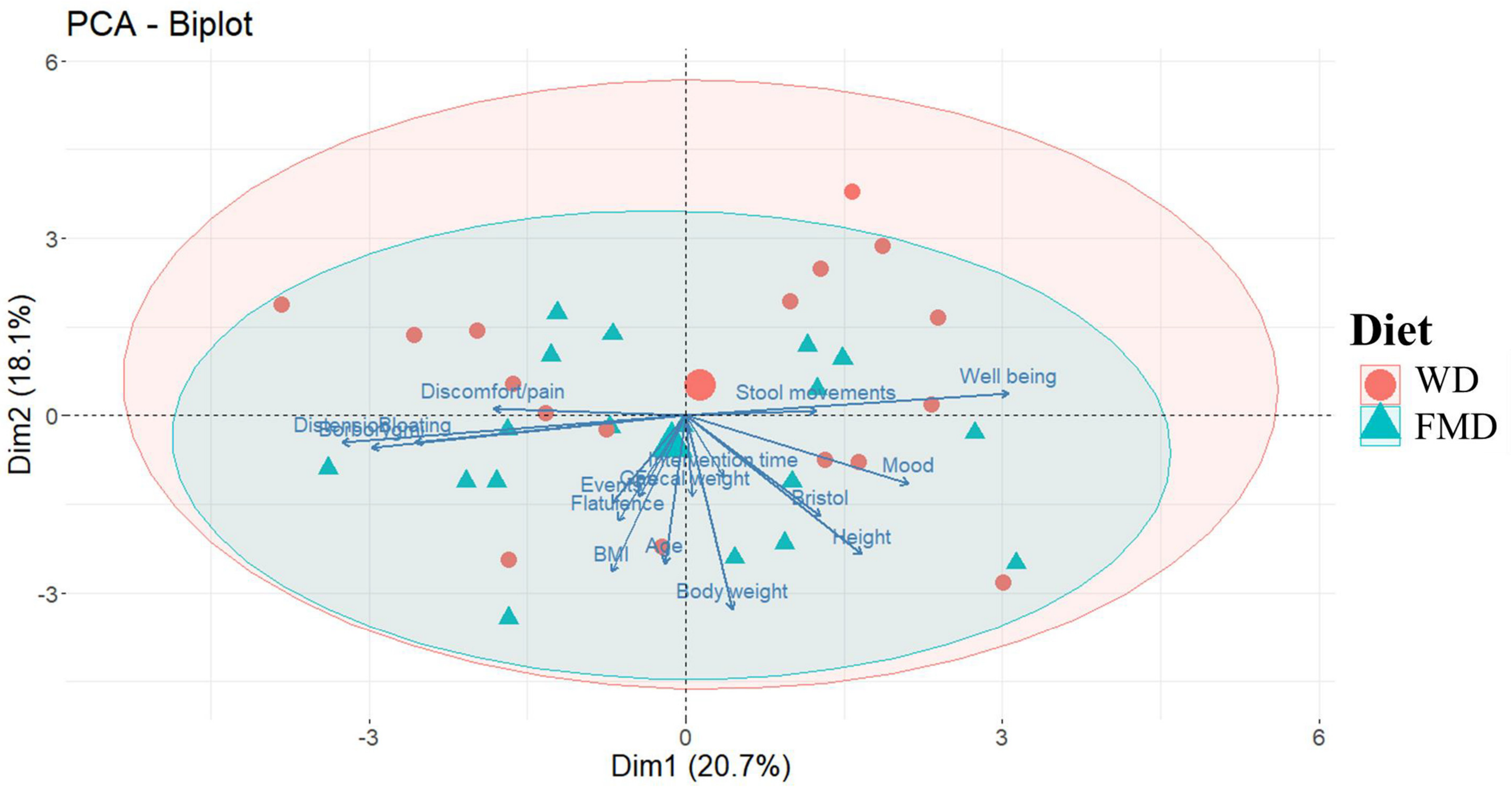

Figure S9. Principal components analysis (**PCA**) of clinical parameters of participants after Western-type Diet (**WD**) or Fiber-enriched Mediterranean-type Diet (**FMD**) interventions. As it can be seen, no relevant differences in clinical symptoms can be observed according to the type of diets administered. **Dim**: dimension (principal component), **BMI**: Body mass index. The percentage of variance explained by each Dim is indicated in the axis.

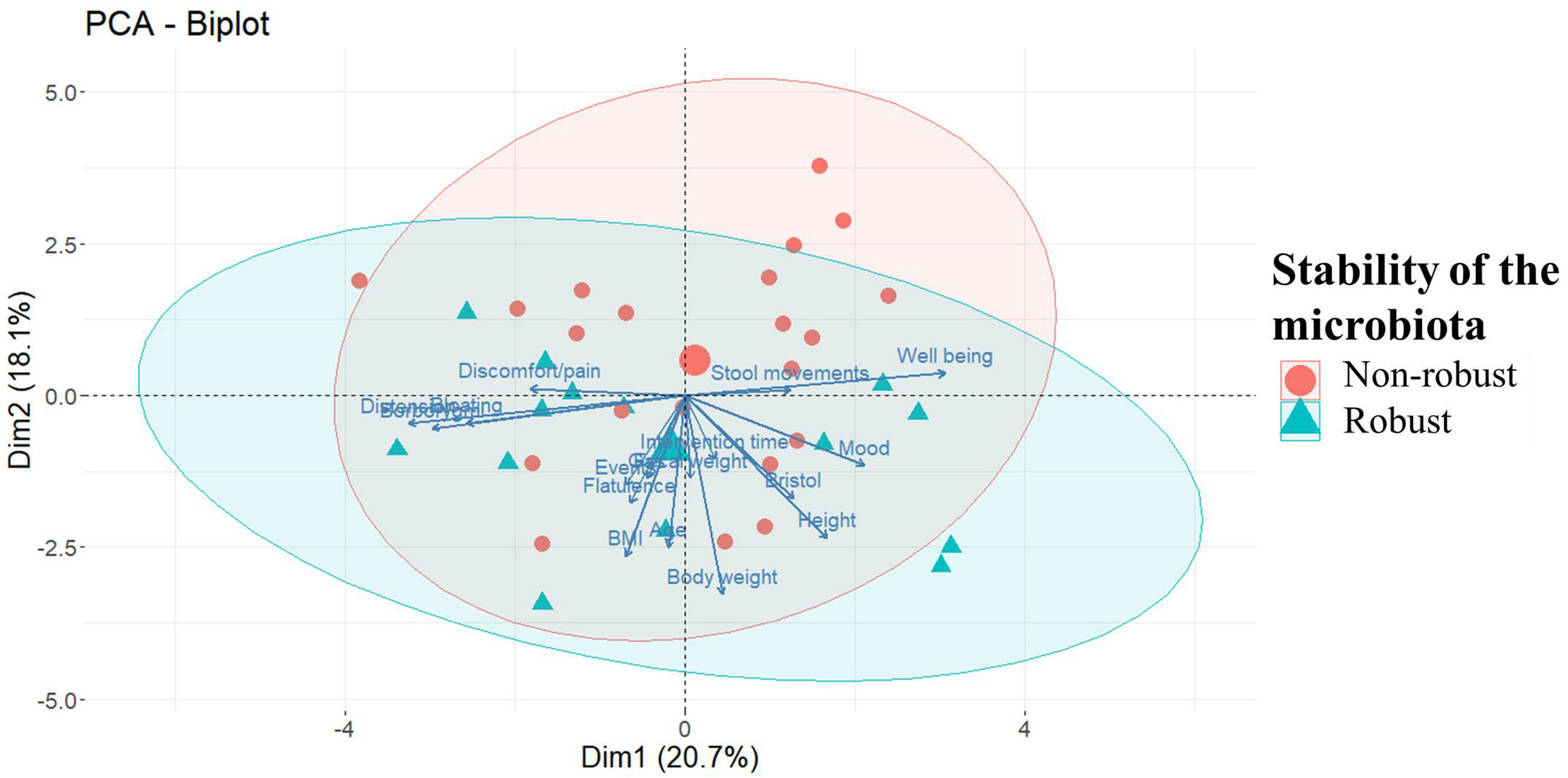

Figure S10. Principal components analysis (**PCA**) of clinical parameters of participants showing a robust and non-robust microbiota (for further details, see **Figure 2**). No relevant differences in clinical symptoms can be observed according to the stability of the microbiota. **Dim**: dimension (principal component), **BMI**: Body mass index. The percentage of variance explained by each Dim is indicated in the axis.

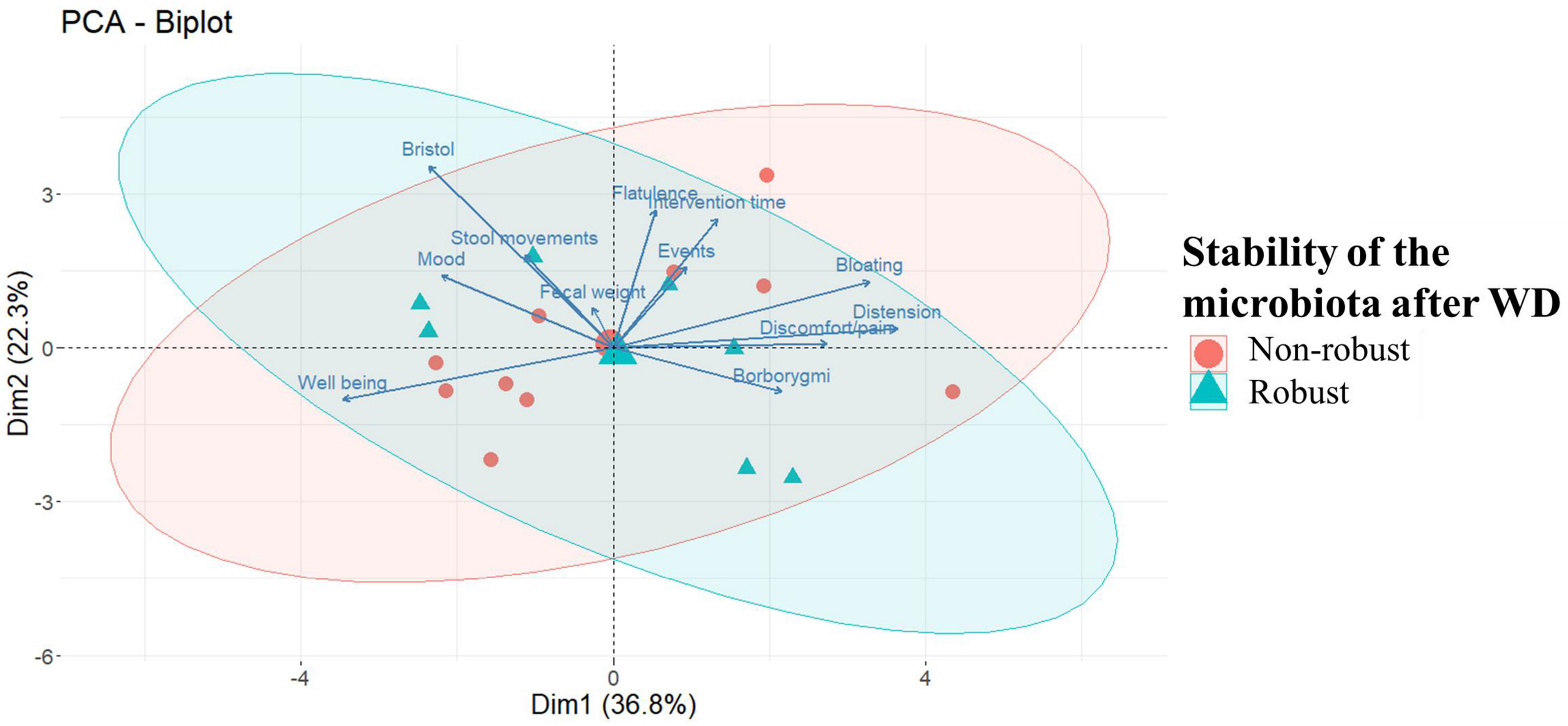

Figure S11. Principal components analysis (**PCA**) of clinical parameters of participants showing a robust and non-robust microbiota after Western-type Diet (**WD**) intervention (for further details, see **Figure 2**). No relevant differences in clinical symptoms can be observed according to the stability of the microbiota. **Dim**: dimension (principal component), **BMI**: Body mass index. The percentage of variance explained by each Dim is indicated in the axis.

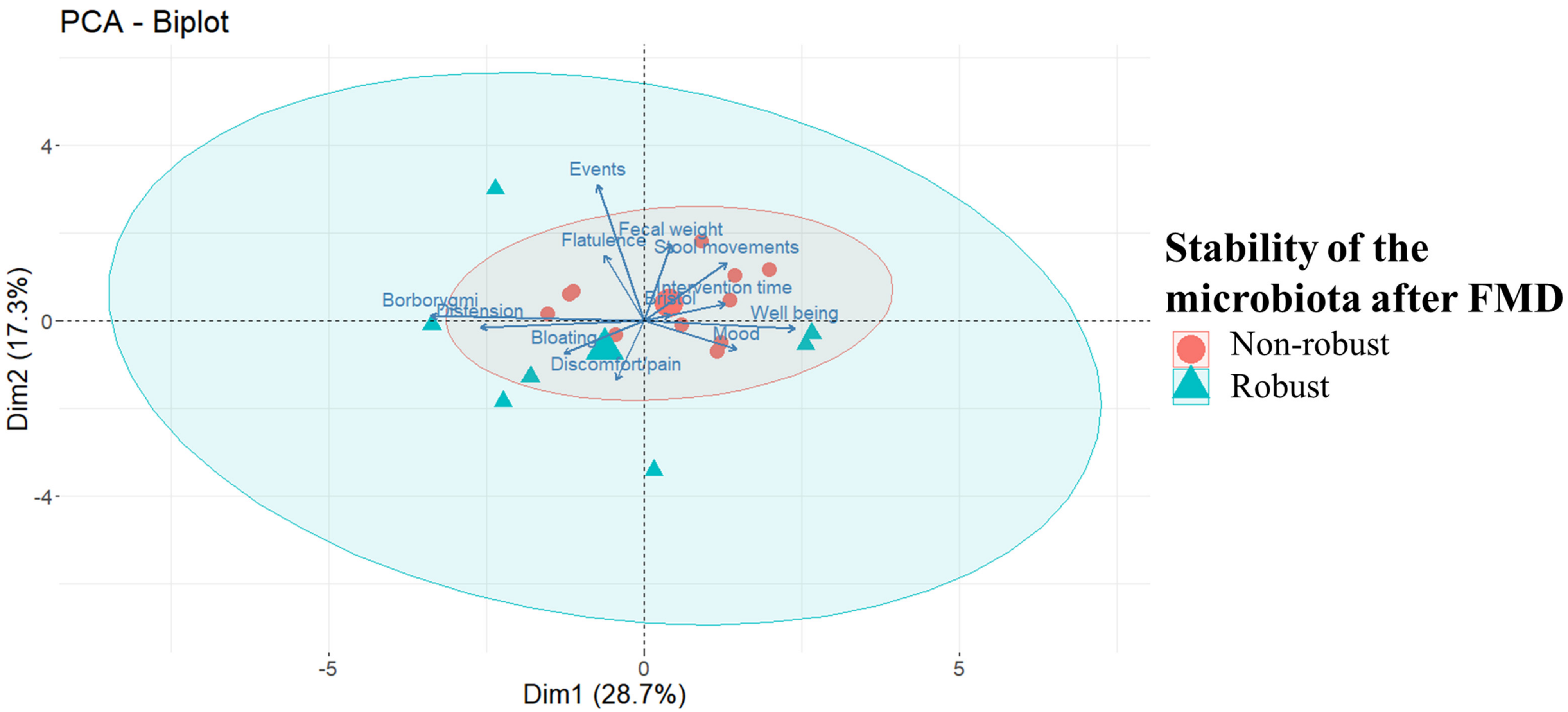

Figure S12. Principal components analysis (**PCA**) of clinical parameters of participants showing a robust and non-robust microbiota after Mediterranean-type Diet (**FMD**) intervention (for further details, see **Figure 2**). No relevant differences in clinical symptoms can be observed according to the stability of the microbiota. **Dim**: dimension (principal component), **BMI**: Body mass index. The percentage of variance explained by each Dim is indicated in the axis.

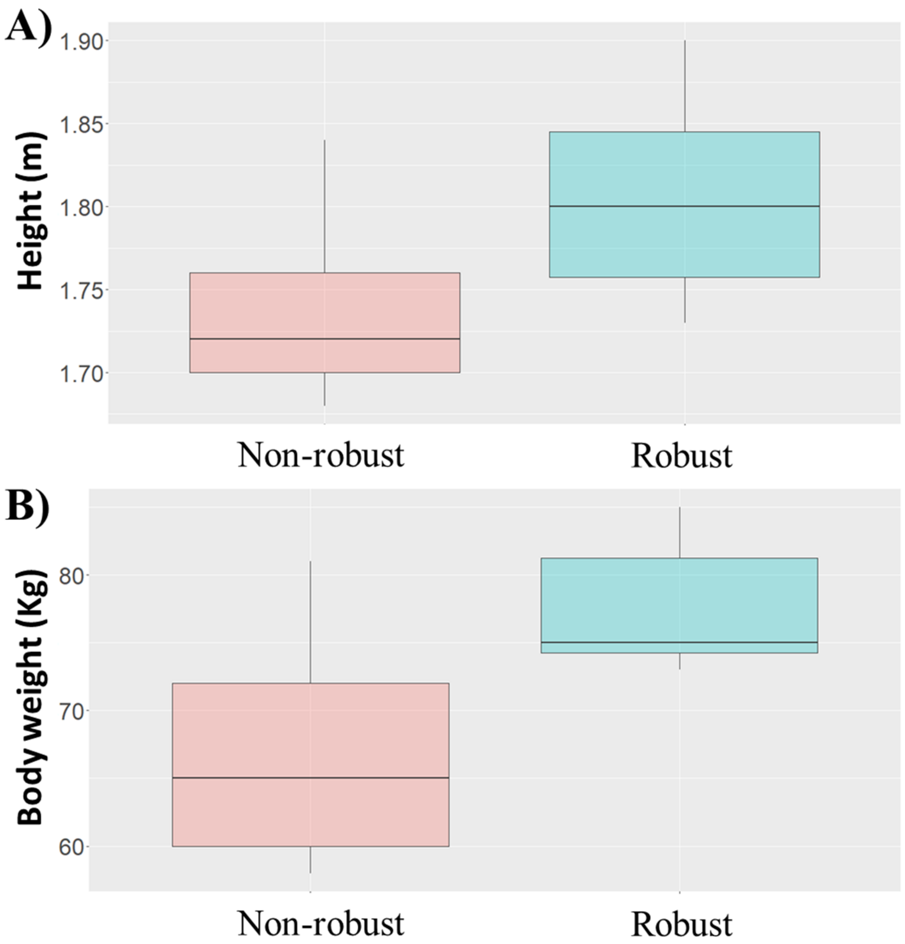

Figure S13. Influence of height and body weight on the stability of the microbiota (for further details, see **Figure 2**). Participants showing higher height and body weight also showed higher microbiota stability after Western-type Diet (**WD**) and Fiber-enriched Mediterranean-type Diet (**FMD**) interventions.

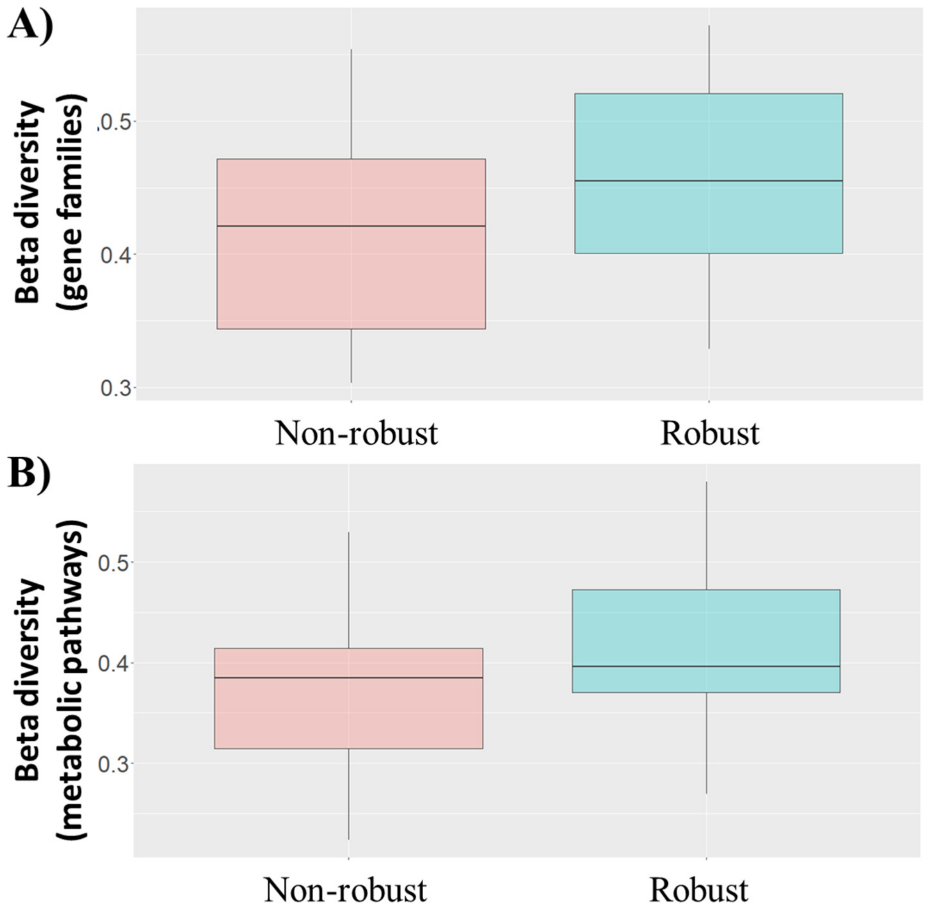

Figure S14. Beta-diversity analysis of gene families (**A**) metabolic pathways (**B**) determined in samples from participants showing a robust and non-robust microbiota (for further details, see **Figure 2**). The Bray-Curtis method was selected for the calculation. Beta-diversity values were slightly higher in robust microbiotas that did not suffer any relevant change after Western-type Diet (**WD**) and Fiber-enriched Mediterranean-type Diet (**FMD**) interventions.
